# MAVS Cys508 palmitoylation promotes its aggregation on the mitochondrial outer membrane and antiviral innate immunity

**DOI:** 10.1073/pnas.2403392121

**Published:** 2024-08-14

**Authors:** Yinong Liu, Dan Hou, Wenzhe Chen, Xuan Lu, Garrison P. Komaniecki, Yilai Xu, Tao Yu, Sophia M. Zhang, Maurine E. Linder, Hening Lin

**Affiliations:** ^a^Department of Chemistry and Chemical Biology, Cornell University, Ithaca, NY 14853; ^b^Department of Molecular Medicine, Cornell University, Ithaca, NY 14853; ^c^Howard Hughes Medical Institute, Department of Chemistry and Chemical Biology, Department of Molecular Biology and Genetics, Cornell University, Ithaca, NY 14853

**Keywords:** palmitoylation, antiviral innate immunity, MAVS, ZDHHC7, tail-anchored proteins

## Abstract

Cysteine palmitoylation or *S*-palmitoylation catalyzed by the ZDHHC family of acyltransferases regulates the biological function of numerous mammalian proteins as well as viral proteins. However, understanding of the role of *S*-palmitoylation in antiviral immunity against RNA viruses remains very limited. The adaptor protein MAVS forms functionally essential prion-like aggregates upon activation by viral RNA-sensing RIG-I-like receptors. Here, we identify that MAVS, a C-terminal tail-anchored mitochondrial outer membrane protein, is *S*-palmitoylated by ZDHHC7 at Cys508, a residue adjacent to the tail-anchor transmembrane helix. Using superresolution microscopy and other biochemical techniques, we found that the mitochondrial localization of MAVS at resting state mainly depends on its transmembrane tail-anchor, without regulation by Cys508 *S*-palmitoylation. However, upon viral infection, MAVS *S*-palmitoylation stabilizes its aggregation on the mitochondrial outer membrane and thus promotes subsequent propagation of antiviral signaling. We further show that inhibition of MAVS *S*-palmitoylation increases the host susceptibility to RNA virus infection, highlighting the importance of *S*-palmitoylation in the antiviral innate immunity. Also, our results indicate ZDHHC7 as a potential therapeutic target for MAVS-related autoimmune diseases.

## Significance

This study established that *S*-palmitoylation catalyzed by ZDHHC7 plays an important role in RIG-I-like receptor signaling and antiviral innate immunity. Mechanistically, the study shows that under resting conditions, *S*-palmitoylation does not affect the mitochondrial localization of the tail-anchored MAVS protein. However, under activating conditions, *S*-palmitoylation is important for stabilizing the aggregation of MAVS on the mitochondrial outer membrane. Many integral membrane proteins are known to be *S*-palmitoylated, but the exact function of *S*-palmitoylation is not very well understood. This finding here provides important insights into how *S*-palmitoylation could affect tail-anchored proteins or integral membrane proteins. The study also indicates that targeting ZDHHC7 or MAVS Cys508 could be an effective strategy to tune immune signaling and control inflammation.

Innate immunity provides the first line of defense, protecting the host from invading pathogens including bacteria, fungi, viruses, and parasites. Among these pathogens, RNA viruses, such as influenza, coronavirus, and HIV, which evolve rapidly and have the potential to cause widespread infection outbreaks, can be sensed by retinoic acid-inducible gene I (RIG-I) and melanoma differentiation-associated gene 5 (MDA5) (1, 2). Both RIG-I and MDA5 can initiate antiviral immune signaling through activation of the downstream adaptor protein MAVS, mitochondrial antiviral-signaling protein (also known as IPS-I/VISA/CARDIF) (3–6). MAVS plays a central role in the RIG-I-like receptor (RLR) signaling pathway, forming prion-like aggregates on the mitochondrial outer membrane (MOM) upon interaction with RIG-I or MDA5 (7). MAVS aggregates serve as a platform for the recruitment of kinases TBK1/IKKε and IKKα/β to activate transcriptional factors, IRF1/IRF3/IRF7 and NF-κB, respectively, which translocate into the nucleus and lead to the expression of interferons (IFNs), proinflammatory cytokines, and other antiviral effector proteins (8–11).

MAVS contains a caspase activation and recruitment domain (CARD) that is essential for RLR-initiated formation of the three-stranded helical MAVS filaments (12), a proline-rich region (PRR) that contributes to the recruitment of tumor necrosis factor receptor-related factors (TRAFs), and a C-terminal transmembrane (TM) domain as the tail anchor to ensure the localization on the MOM and the peroxisomes (3, 11, 13). MiniMAVS, a variant of MAVS that lacks the 141 N-terminal residues containing the CARD and more than half of the PRR, serves as an antagonist of MAVS without interfering with its aggregation (14). In addition to the complex regulation by MAVS-associated proteins (15, 16) and multiple truncated isoforms (17), posttranslational modifications of MAVS have been shown to play a pivotal role in antiviral signaling. For example, phosphorylation of MAVS at different sites triggers different signaling events: Ser442 phosphorylation is essential for IRF3 activation (8), and in contrast, Ser121, Ser212, Ser258, and Ser329 phosphorylation by Nemo-like kinase (NLK) results in MAVS degradation (18). K48-linked ubiquitination leads to MAVS degradation thus down-regulating signaling, whereas K63-linked ubiquitination facilitates MAVS aggregation and K27-linked ubiquitination promotes MAVS interaction with TBK1, resulting in enhanced signaling (19). Besides K63-linked ubiquitination, protein modifications such as poly-SUMOylation and arginine methylation also affect MAVS aggregation (20–22).

*S*-palmitoylation is the covalent attachment of long-chain fatty acyl groups to proteins on cysteine side chains via thioester bonds. *S*-palmitoylation is catalyzed by the ZDHHC family of *S*-acyltransferases and can be removed by deacylases including palmitoyl protein thioesterases (PPTs), acyl protein thioesterases (APTs), and α/β-hydrolase domain (ABHD) proteins (23). *S*-palmitoylation occurs on a diverse set of proteins. Even viral proteins, such as vesicular stomatitis virus (VSV) G protein (24) and severe acute respiratory syndrome coronavirus 2 (SARS-CoV-2) spike protein (25), are palmitoylated by host ZDHHCs, which enhances virus infectivity. *S*-palmitoylation is involved in many physiologically important immune and inflammation processes (26), as recently demonstrated in *S*-palmitoylation studies of stimulator of interferon genes protein (STING) (27), nucleotide-binding oligomerization domain proteins 1/2 (NOD1/2) (28), signal transducer and activator of transcription 3 (STAT3) (29), and nucleotide-binding domain, leucine-rich-containing family, pyrin domain-containing-3 (NLRP3) (30, 31). Mechanistically, *S*-palmitoylation, which increases the hydrophobicity of the modified domain, typically promotes protein localization to membranes and regulates protein trafficking, which in turn could affect protein stability and protein–protein interactions (23). However, the function of *S*-palmitoylation of tail-anchored and integral membrane proteins, especially for tail-anchored MOM proteins, is less understood.

In this study, we found that MAVS is *S*-palmitoylated on Cys508 and the modification is catalyzed by ZDHHC7, and to a smaller extent, by ZDHHC3. This *S*-palmitoylation occurs adjacent to the tail-anchor. Interestingly, Cys508 palmitoylation does not regulate MAVS mitochondrial outer membrane localization at resting state; however, it stabilizes MAVS aggregation on the MOM upon virus infection, therefore promoting RLR signaling and innate antiviral immunity. Our work reveals the molecular function of *S*-palmitoylation of a mitochondrial tail-anchored membrane protein and the physiological role of ZDHHC7 in promoting antiviral immune responses.

## Results

### MAVS Is *S*-Palmitoylated by ZDHHC7.

**Fig. 1.**
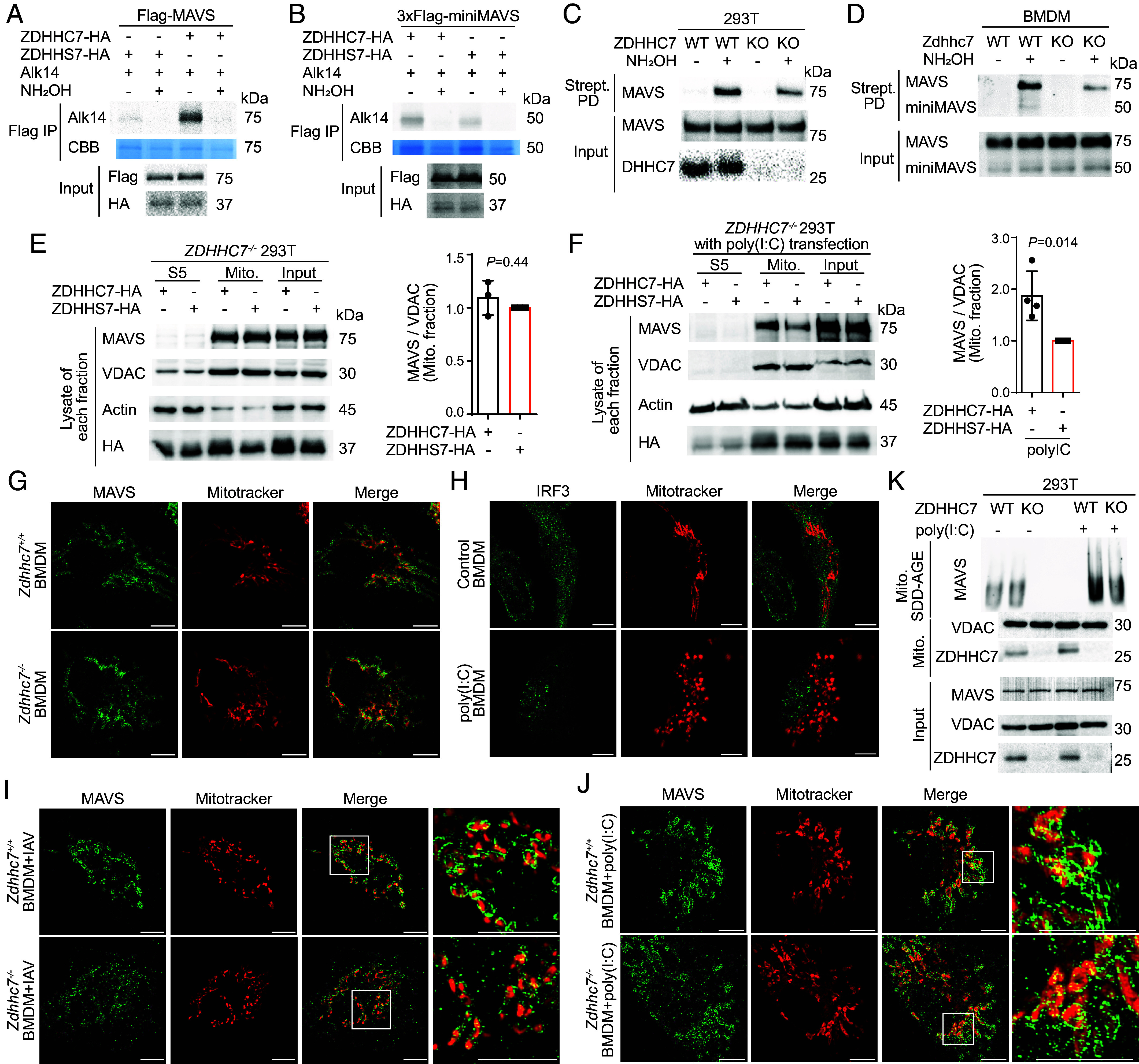
ZDHHC7-catalyzed *S*-palmitoylation stabilizes MAVS aggregation. (*A*) ZDHHC7 overexpression increased MAVS *S*-palmitoylation. HEK293T cells transfected with Flag-MAVS and HA-ZDHHC7 or the catalytic dead mutant, HA-ZDHHS7, were metabolically labeled with 50 μM Alk14 for 6 h. Flag-MAVS was immunoprecipitated from cell lysate and fluorescently labeled via click chemistry. The palmitoylation signal with or without hydroxylamine (NH_2_OH) treatment was detected by in-gel fluorescence. IP: immunoprecipitation. CBB: Coomassie Brilliant Blue. (*B*) MiniMAVS was *S*-palmitoylated. HEK293T cells transfected with 3xFlag-miniMAVS and HA-ZDHHC7 or HA-DHHS7 were metabolically labeled with 50 μM Alk14 for 6 h. The palmitoylation signal with or without NH_2_OH treatment was detected by in-gel fluorescence. (*C*) ZDHHC7 knockout in HEK293T cells decreased endogenous MAVS *S*-palmitoylation. *S*-palmitoylation was detected using the ABE assay. Strept PD: Streptavidin pull down. See quantification in *SI Appendix*, Fig. S1*E*, *n* = 5. Strept PD signal on the immunoblots represented 50% of MAVS *S*-acylation level and the input represented 5% of total MAVS protein. (*D*) ZDHHC7 knockout in BMDMs decreased endogenous MAVS *S*-palmitoylation. *S*-palmitoylation was detected using the ABE assay. (*E*) At resting state, ZDHHC7 did not affect MAVS mitochondrial localization. *ZDHHC7^−/−^* HEK293T cells ectopically expressing HA-ZDHHC7 or HA-ZDHHS7 were subjected to subcellular fractionation to analyze MAVS level in supernatant 5 (S5), crude mitochondria pellet 5 (P5), and input. The relative levels of MAVS in the mitochondrial fraction were quantified (right). Number of independent experiments, n = 3. (*F*) Under poly(I:C) activation, ZDHHC7 affected MAVS mitochondrial localization. *ZDHHC7^−/−^* 293 T cells ectopically expressing HA-ZDHHC7 or HA-ZDHHS7 were transfected with 2 μg/mL poly(I:C) for 8 h and then subjected to subcellular fractionation. The distribution of MAVS and the mitochondria marker VDAC were analyzed by immunoblotting. The relative levels of MAVS in the mitochondrial fraction were quantified and shown on the right. Number of independent experiments, n = 4. (*G*) At resting state, the mitochondrial localization of MAVS was not affected by ZDHHC7. Endogenous MAVS (green) and mitochondria (red) in nonstimulated WT and *Zdhhc7^−/−^* BMDMs were visualized using SR-SIM (Scale bars, 5.0 μm.) (*H*) Poly(I:C) stimulation induced mitochondrial fragmentation. Distribution of IRF3 (green) and morphology of the mitochondria of nonstimulated or poly(I:C)-transfected BMDMs were imaged using SR-SIM (Scale bars, 5.0 μm.) (*I*) Under IAV infection, ZDHHC7 affected MAVS aggregation on the mitochondrial outer membrane. WT and *Zdhhc7^−/−^* BMDMs were infected with IAV for 8 h. Endogenous MAVS (green) and mitochondria (red) were visualized by SR-SIM (Scale bars, 5.0 μm.) See quantification of the size of MAVS aggregation in *SI Appendix*, Fig. S3*H*. (*J*) Under poly(I:C) stimulation, ZDHHC7 affected MAVS aggregation on the mitochondrial outer membrane. WT and *Zdhhc7^−/−^* BMDMs were stimulated by 1 μg/mL poly(I:C) transfection for 6 h. Endogenous MAVS (green) and mitochondria (red) were visualized by SR-SIM (Scale bars, 5.0 μm.) (*K*) SDD-AGE result revealed that ZDHHC7 promotes MAVS aggregation under stimulation. WT and *ZDHHC7^−/−^* HEK293T cells treated with or without poly(I:C) at 2 μg/mL for 8 h by transfection were collected for subcellular fractionation. SDD-AGE was used to detect MAVS aggregation in the crude mitochondria P5 fractions. Data are mean ± SD in *E* and *F*. *P* values were calculated using two-tailed ratio paired *t* tests.

Activation of multiple adaptor proteins in immune signaling pathways, including STAT3, STING, and MYD88, is strongly affected by *S*-palmitoylation (27, 29, 32). Given that MAVS serves as a central adaptor protein in the RLR pathway, we wondered whether the activation of MAVS is regulated by *S*-palmitoylation. To examine whether MAVS is *S*-palmitoylated, we first employed metabolic labeling with alkyne-tagged palmitic acid (Alk14) (29) and click chemistry conjugation of TAMRA-azide to detect the palmitoylation of MAVS. In-gel fluorescence results showed that MAVS was modified by Alk14, and expression of the *S*-palmitoyltransferase ZDHHC7 significantly enhanced the modification. Treatment of hydroxylamine (NH_2_OH), which cleaves thioesters but not amide or oxyester bonds, largely removed the Alk14 labeling signal of MAVS, indicating that the modification occurred at cysteine residue(s) (Fig. 1*A*). Similarly, miniMAVS, the N-terminal truncated isoform of MAVS, which only contains the last four cysteines of full-length MAVS, was labeled by Alk14 and the labeling was NH_2_OH sensitive (Fig. 1*B*), suggesting that miniMAVS is also *S*-palmitoylated. Next, we demonstrated that endogenous MAVS is *S*-acylated using the acyl-biotin exchange (ABE) assay, in which unmodified cysteines were blocked, thioester modifications were removed by NH_2_OH, and the released free cysteines (the previous *S*-acylation sites) were biotinylated for pull-down (29). Endogenous *S*-acylation of MAVS was detected in A549 cells, and the level was not affected by the stimulation of RLR signaling using polyinosinic-polycytidylic acid (poly(I:C)), a synthetic analog of double-stranded RNA (dsRNA) that mimics viral RNA to trigger the RLR signaling, or influenza A virus (IAV, H1N1 strain A/PR/8/34) infection (*SI Appendix*, Fig. S1*A*) (33). Moreover, treatment of 2-bromopalmitate (2-BP) (34), a pan-ZDHHC inhibitor, reduced endogenous MAVS *S*-acylation level in HEK293T cells, indicating that MAVS *S*-acylation is ZDHHC-dependent (*SI Appendix*, Fig. S1*B*).

To systematically identify the *S*-palmitoyltransferase(s) of MAVS, we screened the 23 ZDHHC enzymes (23) using Alk14 labeling of Flag-MAVS. Among the 23 ZDHHCs, we found that only the expression of ZDHHC3 and ZDHHC7 substantially increased MAVS palmitoylation by 3-fold and 4-fold, respectively (*SI Appendix*, Fig. S1 *C* and *D*). Overexpression of ZDHHC4, previously identified as a MAVS interacting protein (35), and ZDHHC8, localized in the mitochondria (36), did not increase MAVS palmitoylation (*SI Appendix*, Fig. S1 *C* and *D*). More importantly, MAVS *S*-palmitoylation levels were significantly decreased in *ZDHHC7^−/−^* (Fig. 1*C* and *SI Appendix*, Fig. S1*E*) and *ZDHHC3^−/−^* HEK293T cells (*SI Appendix*, Fig. S1*F*) compared to WT cells, supporting that MAVS is a substrate of ZDHHC3 and ZDHHC7 under physiological conditions. Moreover, ZDHHC7 knockdown in *ZDHHC3^−/−^* HEK293T cells further reduced the level of MAVS *S*-palmitoylation to 12% of that in WT cells (*SI Appendix*, Fig. S1*G*), suggesting that ZDHHC3 and ZDHHC7 are the two major palmitoyltransferases of MAVS. We further confirmed that *Zdhhc7* knockout (KO) reduced MAVS *S*-palmitoylation in primary bone marrow–derived macrophages (BMDMs) isolated from WT and *Zdhhc7^−/−^* mice (Fig. 1*D*). Additionally, MAVS coimmunoprecipitated (co-IP) ZDHHC7 in the phorbol 12-myristate 13-acetate (PMA) differentiated THP-1 macrophages, demonstrating their direct physical interaction (*SI Appendix*, Fig. S1*H*). For further studies, we focused on ZDHHC7.

To identify whether *S*-acylation of MAVS is also dynamically regulated by depalmitoylase(s), we treated the cells with depalmitoylase inhibitors, ML348 (an APT1 inhibitor), ML349 (an APT2 inhibitor), and Palmostatin B (a pan-depalmitoylase inhibitor). (37) MAVS palmitoylation was not affected by treatment of these inhibitors (*SI Appendix*, Fig. S2 *A*–*C*). Palmitoyl-protein thioesterase 1 (PPT1) removed MAVS palmitoylation under overexpression; however, MAVS palmitoylation was not affected by knock-down of *PPT1* (*SI Appendix*, Fig. S2 *D*–*E*). These data suggest that MAVS palmitoylation is likely not regulated by APTs, PPTs, or ABHDs under normal conditions.

### ZDHHC7 Promotes Aggregation of Activated MAVS on the Mitochondria.

Protein lipidation typically promotes protein–membrane associations. MAVS is a C-terminal tail-anchored protein on the mitochondrial outer membrane and forms prion-like aggregation upon activation. Therefore, we investigated the effect of palmitoylation on MAVS subcellular localization and aggregation. Using subcellular fractionation, we found that at resting state, reintroduction of ZDHHC7 in *ZDHHC7*^−/−^ HEK293T cells did not exhibit any overt effect on the mitochondrial association of MAVS compared to reintroduction of a catalytically dead mutant ZDHHS7 (Fig. 1*E*). We reasoned that under conditions that MAVS forms aggregates, MAVS palmitoylation may become more important. Indeed, when RLR signaling was stimulated by poly(I:C) transfection, ZDHHC7 overexpression enhanced the mitochondrial association of MAVS in HEK293T cells (*SI Appendix*, Fig. S3*A*). Similarly, reintroducing ZDHHC7 into *ZDHHC7^−/−^* HEK293T cells rescued the amounts of MAVS in the mitochondria fraction with poly(I:C) (Fig. 1*F*). In addition, inhibition of MAVS palmitoylation by 2-BP treatment reduced its mitochondrial localization (*SI Appendix*, Fig. S3*B*). Given that the crude mitochondria fraction contains the mitochondria, the mitochondria-associate membrane (MAM, part of ER membranes), and the peroxisome, we did a Percoll gradient fractionation (38) to further purify MAM and mitochondria (*SI Appendix*, Fig. S3*C*). We found that MAVS localized on both MAM and mitochondria, as previously reported (38). With poly(I:C) activation, reintroducing ZDHHC7 into *ZDHHC7^−/−^* HEK293T cells did not affect the level of MAVS on the MAM but increased its level on the mitochondria, suggesting that when activated, palmitoylation facilitates MAVS mitochondrial localization without affecting the MAM localized MAVS (*SI Appendix*, Fig. S3*D*).

To explain how *S*-palmitoylation affects the mitochondrial localization only for activated MAVS, we hypothesized that palmitoylation actually promotes and stabilizes MAVS aggregates on the mitochondrial outer membrane. Although confocal microscopy images revealed an enhanced mitochondrial localization and intensity of MAVS upon ZDHHC7 overexpression, MAVS aggregates were not resolved (*SI Appendix*, Fig. S3*E*). To resolve MAVS aggregates under different conditions, we first performed immunofluorescence imaging using superresolution structured illumination microscopy (SR-SIM) in A549 cells (12). Redistribution and condensation of MAVS around the mitochondria were observed after IAV infection (*SI Appendix*, Fig. S3*F*), forming aggregates with a maximum filament length of 750 nm. To investigate the role of ZDHHC7 in MAVS aggregation, we compared SR-SIM images of MAVS in WT and *Zdhhc7^−/−^* primary BMDMs at resting state and after RLR signaling activation. Without IAV infection, MAVS was distributed relatively evenly surrounding the MitoTracker signal in both WT and *Zdhhc7^−/−^* BMDMs (Fig. 1*G*), which is consistent with its localization on the mitochondrial outer membrane. Under stimulation with poly(I:C), the activated cells, as determined by the enriched IRF3 in the nucleus, showed significant fragmentation of fused mitochondria, which can be considered as an indicator of RLR activation (Fig. 1*H* and *SI Appendix*, Fig. S3*G*). With IAV infection, MAVS formed brighter and more intense aggregates concentrated on the mitochondria in WT BMDMs. In contrast, in *Zdhhc7^−/−^* BMDMs, MAVS formed much smaller and more dispersed aggregates on the mitochondria, and some of the aggregates were not associated with mitochondria (Fig. 1*I* and *SI Appendix*, Fig. S3*H*). Similar patterns of MAVS distribution were also observed when comparing poly(I:C)-stimulated WT and *Zdhhc7^−/−^* BMDMs (Fig. 1*J*). Biochemically, semidenaturing detergent agarose-gel electrophoresis (SDD-AGE) of the crude mitochondria P5 fraction showed that *ZDHHC7* KO suppressed MAVS aggregation in the poly(I:C)-stimulated HEK293T cells (Fig. 1*K*). Collectively, both the superresolution imaging and biochemical evidence suggest that ZDHHC7 promotes and stabilizes MAVS aggregation on the mitochondria outer membrane under RLR activation conditions.

### ZDHHC7 Palmitoylates MAVS C508 to Enhance its Aggregation on the MOM.

**Fig. 2.**
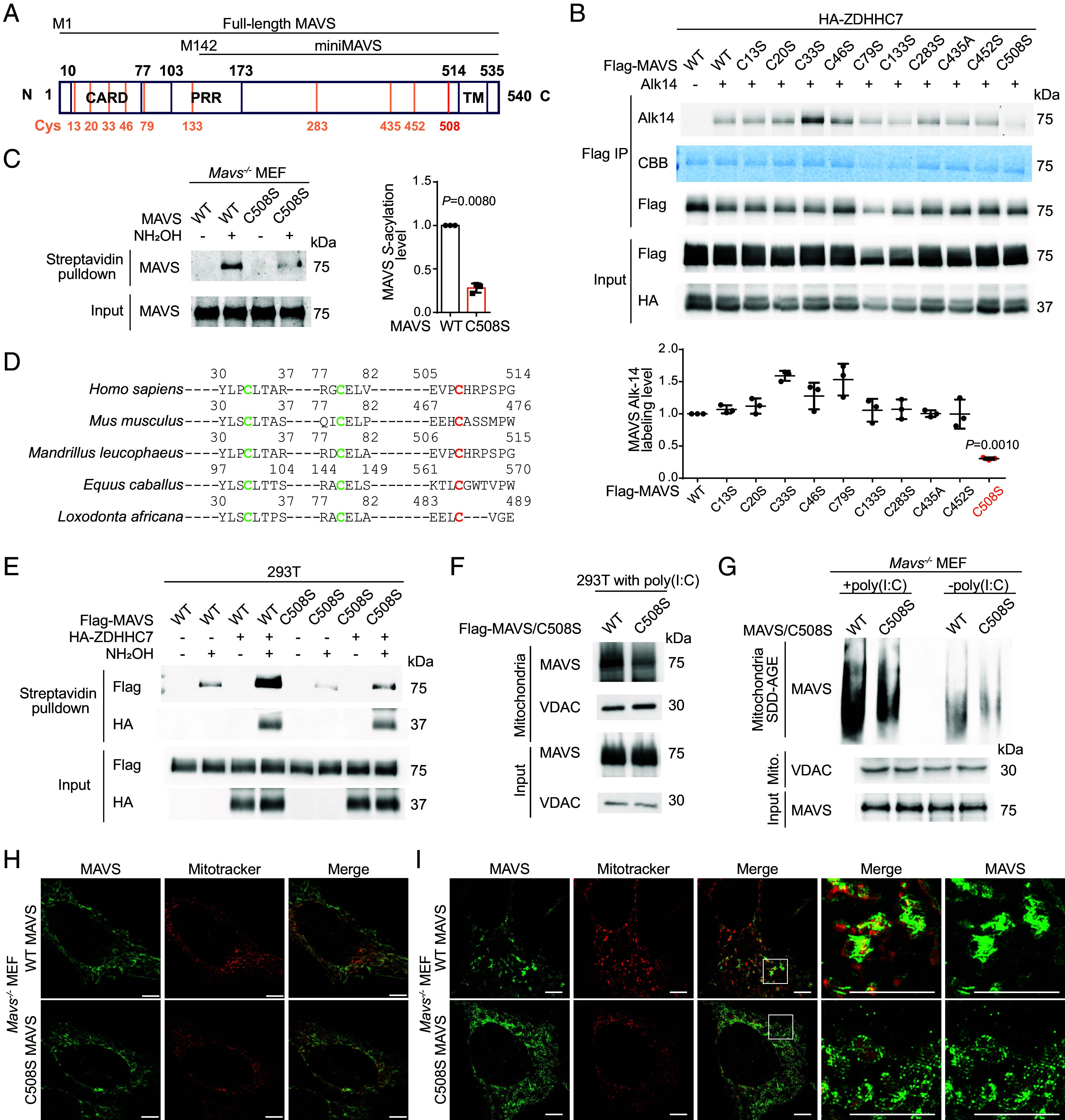
MAVS C508 palmitoylation promotes its aggregation on the mitochondria. (*A*) A diagram showing domains of MAVS and miniMAVS. Ten cysteine residues of full-length MAVS are shown in orange. CARD: caspase activation and recruitment domain. PRR: Proline-rich region. TM: transmembrane domain. (*B*) Identification of the *S*-palmitoylation site of MAVS by mutagenesis. HEK293T cells were transfected with WT Flag-MAVS or indicated Cys-to-Ser or Cys-to-Ala mutant and HA-ZDHHC7, labeled with 50 μM Alk14 for 6 h, and palmitoylation was detected by in-gel fluorescence after Flag-MAVS immunoprecipitation and click chemistry. Number of independent experiments, n = 3. (*C*) ABE assay result showing the *S*-acylation level of WT or C508S MAVS reintroduced into *Mavs^−/−^* MEF cells. Quantification of results from three independent experiments is shown on the right. Number of independent experiments, n = 3. (*D*) Sequence alignment of MAVS from different species. Orange: identified palmitoylated site. Green: two other conserved cysteines. (*E*) ABE assay result showing the *S*-acylation level of Flag-MAVS WT or C508S mutant in HEK293T cells cotransfected with empty vector or HA-ZDHHC7. (*F*) Distribution of MAVS WT or C508S mutant in crude mitochondria P5 fraction of HEK293T cells activated with 1 μg/mL poly(I:C) for 8 h. Flag-MAVS was transfected for 16 h before stimulation. (*G*) Aggregation of MAVS WT or C508S mutant detected by SDD-AGE. *Mavs^−/−^* MEF cells stably expressing MAVS WT or C508S were treated with or without poly(I:C) and the mitochondrial P5 fractions were obtained and analyzed by SDD-AGE to reveal MAVS aggregation. (*H*) Subcellular distribution of MAVS (green) and mitochondria (red) in nonstimulated *Mavs^−/−^* MEF cells stably expressing MAVS WT or C508S mutant. The images were obtained using SR-SIM (Scale bars, 5.0 μm.) (*I*) Subcellular distribution of MAVS (green) and mitochondria (red) in poly(I:C) transfected *Mavs^−/−^* MEF cells stably expressing WT or C508S MAVS. The images were obtained using SR-SIM (Scale bars, 5.0 μm.) Data are mean ± SD in *B* and *C*. *P* values were calculated using two-tailed ratio paired *t* tests.

To further confirm that ZDHHC7 promotes MAVS aggregation on the MOM by promoting its *S*-palmitoylation, we sought to identify the *S*-palmitoylation site of MAVS. We mutated individual cysteine of MAVS to serine (except C435, which is mutated to alanine because the C435S mutant was strongly phosphorylated) (*SI Appendix*, Fig. S4 *A* and *B*) and applied Alk14 metabolic labeling to evaluate their palmitoylation status. Under ZDHHC7 overexpression, the C508S mutant had a significant decrease of Alk14 labeling compared to WT, while other mutants (including MAVS C79F mutation, which alleviates systemic lupus erythematosus symptom due to abolished MAVS aggregation) (39) largely retained the modification (Fig. 2 *A* and *B* and *SI Appendix*, Fig. S4*C*), suggesting that C508 is the predominant palmitoylation site of MAVS catalyzed by ZDHHC7. C508S was also the only mutation that significantly decreased the palmitoylation level of MAVS with ZDHHC3 overexpression (*SI Appendix*, Fig. S4*D*), suggesting that both ZDHHC7 and ZDHHC3 work on the same site. Mutation of C367 of miniMAVS, corresponding to MAVS C508, to serine abolished the palmitoylation (*SI Appendix*, Fig. S4*E*), further supporting that *S*-palmitoylation primarily occurs at C508 of MAVS. We then reintroduced WT or C508S mutant of MAVS to *Mavs^−/−^* MEF cells. The C508S mutant had significantly decreased *S*-palmitoylation level as revealed by ABE assay (Fig. 2*C*), demonstrating that C508 is the predominant *S*-palmitoylation site catalyzed by endogenous ZDHHCs. C508 is close to the C-terminal transmembrane domain of MAVS (Fig. 2*A* and *SI Appendix*, Fig. S4*F*) and might be more accessible for ZDHHCs compared to cysteines in the well-folded CARD (*SI Appendix*, Fig. S4*F*). C508 is conserved in multiple species (Fig. 2*D*). Moreover, ABE assay showed that overexpression of ZDHHC7 in HEK293T cells increased WT MAVS palmitoylation but did not have much effect on the C508S mutant, suggesting that ZDHHC7 specifically acts on C508 of MAVS (Fig. 2*E*).

Next, we investigated whether MAVS C508 palmitoylation affects the subcellular distribution of the activated MAVS condensates. In poly(I:C) stimulated HEK293T cells, WT MAVS colocalized with the mitochondria to a greater extent than the palmitoylation-deficient C508S mutant as revealed by subcellular fractionation (Fig. 2*F*). Moreover, *Mavs^−/−^* MEF cells reexpressing WT MAVS showed more aggregation compared to cells reexpressing the C508S mutant as shown by SDD-AGE (Fig. 2*G*), suggesting that MAVS aggregation on the mitochondria is enhanced by C508 palmitoylation under activated conditions. In line with the finding that ZDHHC7 does not regulate MAVS localization at resting state (Fig. 1*G*), both WT and C508S MAVS were relatively evenly distributed on the surface of mitochondria when not activated (Fig. 2*H*), visualized using SR-SIM imaging. However, when stimulated by poly(I:C), WT MAVS redistributed into intense and rod-like aggregates on the mitochondria, on average 550 nm in length, while such MAVS filaments were not observed for the palmitoylation-deficient C508S mutant (Fig. 2*I*). Instead, C508S MAVS formed small-dot aggregates upon stimulation, and more C508S MAVS disassociated from mitochondria and localized in the cytosol (Fig. 2*I* and *SI Appendix*, Fig. S4*G*). The length of WT MAVS aggregates formed with poly(I:C) activation is comparable with a previous study showing that MAVS formed 400 nm filaments upon Sendai Virus infection (12). In addition, we observed some hyper-MAVS filaments with a length of 800 nm wrapping the mitochondria that were not well-stained by MitoTracker Red (*SI Appendix*, Fig. S4*H*). Since MitoTracker Red also reflects the mitochondria membrane potential (40), this weak staining of mitochondria suggests the possibility that hyperactivated MAVS may induce mitochondria membrane potential loss. Collectively, these data support that MAVS C508 palmitoylation by ZDHHC7 is required for the formation of MAVS filaments on the mitochondrial outer membrane when RLR signaling is activated.

### ZDHHC7 Enhances RLR Signaling and Antiviral Immune Response.

**Fig. 3.**
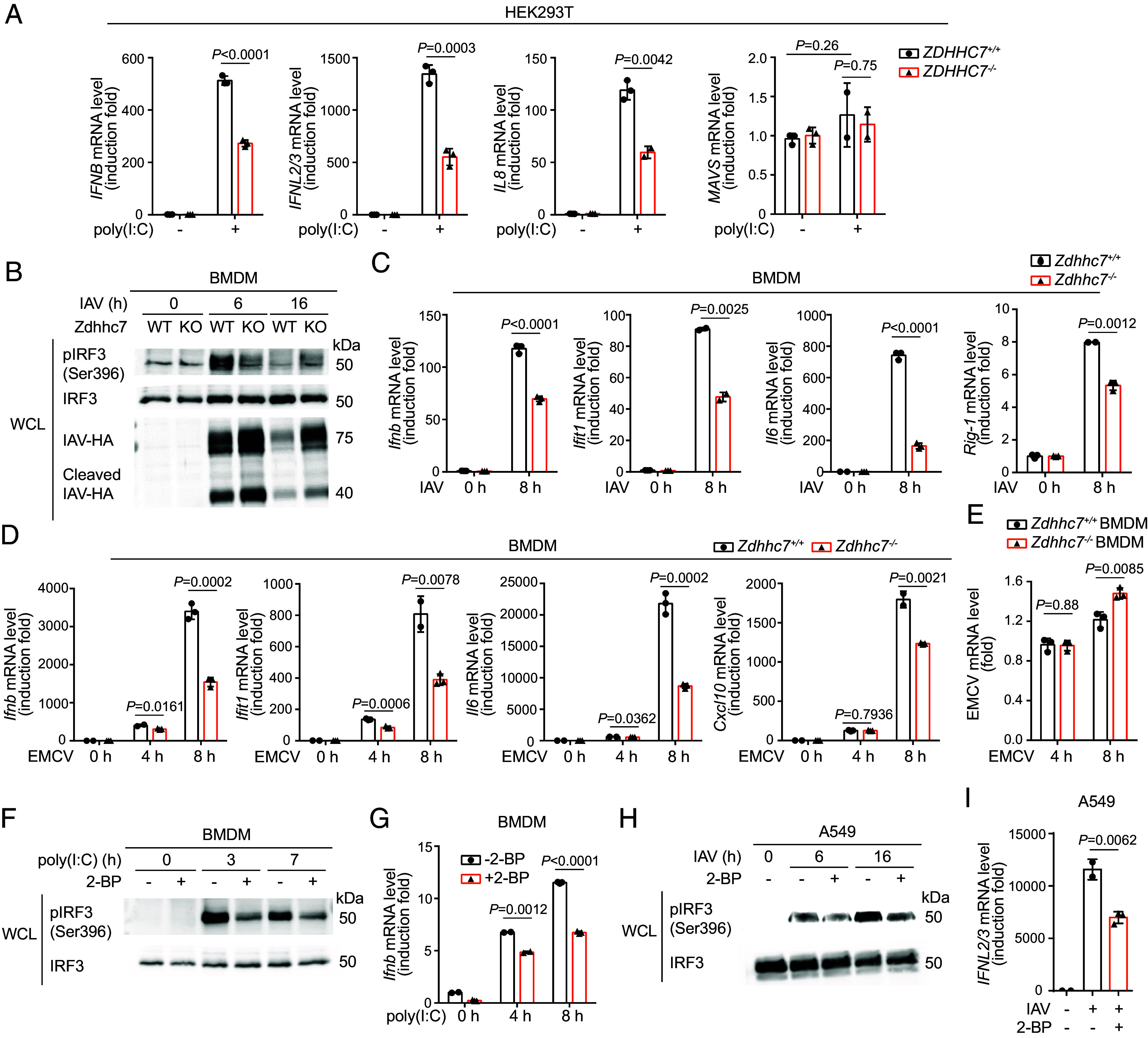
ZDHHC7 enhances RLR signaling and antiviral immune response. (*A*) *ZDHHC7* KO in HEK293T cells decreased poly(I:C)-induced type I and type III interferon and pro-inflammatory cytokine expression. WT and *ZDHHC7^−/−^* HEK293T cells were stimulated with or without 200 ng/mL poly(I:C) transfection for 16 h. Induction of *IFNB*, *IFNL2/3*, *IL8*, and *MAVS* was measured using qPCR. (*B*) *ZDHHC7* KO in BMDMs decreased IAV-induced IRF3 activation. Primary BMDMs isolated from WT or *Zdhhc7^−/−^* mice were cultured and infected with IAV for 0, 6, and 16 h. Whole-cell lysate (WCL) was collected and blotted for pIRF3 (Ser396), IRF3, and IAV hemagglutinin (IAV-HA). (*C*) *ZDHHC7* KO in BMDMs decreased IAV-induced antiviral gene expression. Inductions of *Ifnb*, *Ifit1*, *Il6,* and *Rig-1* in WT or *Zdhhc7^−/−^* primary BMDMs infected with or without IAV was analyzed by qPCR. (*D*) *ZDHHC7* KO in BMDMs decreased EMCV-induced antiviral gene expression. Induction of *Ifnb*, *Ifit1*, *Il6,* and *Cxcl10* in EMCV infected WT or *Zdhhc7^−/−^* primary BMDMs was analyzed by qPCR. (*E*) *Zdhhc7^−/−^* BMDMs exhibited higher EMCV load. EMCV gene was detected by qPCR. (*F*) Inhibition of Zdhhc7 by 2-BP decreased poly(I:C) stimulated IRF3 activation in BMDMs. WT BMDMs were pretreated with 50 μM 2-BP or DMSO for 2 h and stimulated by poly(I:C) for 0, 3, and 7 h. Levels of pIRF3 and IRF3 were determined by immunoblotting. (*G*) Inhibition of Zdhhc7 by 2-BP decreased poly(I:C) stimulated *Ifnb* expression in BMDMs. WT BMDMs were pretreated with 50 μM 2-BP or DMSO for 2 h and stimulated by poly(I:C) for 0, 4, and 8 h. Induction of *Ifnb* was analyzed using qPCR. (*H*) Inhibition of ZDHHC7 by 2-BP decreased IAV-stimulated IRF3 activation in A549 cells. A549 cells were pretreated with 50 μM 2-BP or DMSO for 2 h and infected by IAV for 6 or 16 h. Levels of pIRF3 and IRF3 were determined by immunoblots. (*I*) Inhibition of ZDHHC7 by 2-BP decreased IAV-stimulated *IFNL2/3* expression in A549 cells. Induction of *IFNL2/3* was analyzed by qPCR. mRNA level in each sample was normalized to internal control (*ACTB* or *Actb*). *P* values were calculated using two-tailed unpaired *t* tests. Data are mean ± SD.

As ZDHHC7-mediated MAVS *S*-palmitoylation promotes its aggregation, which is a key feature of its activation, we next investigated whether ZDHHC7 could promote the RLR signaling and anti-RNA virus innate immune response. In HEK293T cells with ectopic expression of MAVS, coexpression of ZDHHC7 enhanced the activation of IRF3 as demonstrated by its increased phosphorylation level (*SI Appendix*, Fig. S5*A*). ZDHHC7 overexpression alone increased the basal level of pIRF3 and further increased pIRF3 when stimulated by poly(I:C) (*SI Appendix*, Fig. S5 *B* and *C*). Moreover, with poly(I:C) transfection, reintroduction of ZDHHC7 promoted the induction of *IFNB* compared to reintroduction of ZDHHS7 in *ZDHHC7^−/−^* HEK293T cells (*SI Appendix*, Fig. S5*D*), suggesting that ZDHHC7 overexpression positively regulates RLR activity. We next evaluated RLR signaling in WT and *ZDHHC7^−/−^* HEK293T cells. While poly(I:C) can also be recognized by endosomal Toll-like receptor 3 (TLR3), the absence of the confounding TLR3 activity in HEK293T ruled out potential impact through the TLR3 pathway, as evidenced by the fact that direct addition (no transfection) of poly(I:C) did not induce *IFNB* (*SI Appendix*, Fig. S5*E*). Upon stimulation by transfected cytosolic poly(I:C), the MAVS palmitoylation deficient *ZDHHC7^−/−^* HEK293T cells showed suppressed induction of *IFNB*, *IFNL2/3*, and *IL8*, with *MAVS* level unchanged (Fig. 3*A* and *SI Appendix*, Fig. S5*F*). Consistent with the biochemical data showing that ZDHHC3 could palmitoylate MAVS, *ZDHHC3^−/−^* HEK293T also decreased the induction of *IFNB* with poly(I:C) (*SI Appendix*, Fig. S5*G*).

To evaluate the involvement of ZDHHC7 in the RLR signaling in primary cells, we first transfected poly(I:C) into WT and *Zdhhc7^−/−^* primary MEF cells. *Zdhhc7^−/−^* primary MEF cells showed reduced activation of IRF3 and induction of *Ifnb* and *Il6* (*SI Appendix*, Fig. S5 *H*–*J*). To further examine the role of ZDHHC7 in the antiviral effect in primary cells, we infected WT and *Zdhhc7^−/−^* BMDMs with influenza A virus H1N1 strain A/PR/8/34 and monitored the activation of IRF3 (pIRF3). The activation of IRF3 was dependent on IAV-multiplicity of infection (MOI), and *Zdhhc7^−/−^* BMDMs had weaker response in both low and high MOI conditions (*SI Appendix*, Fig. S5*K*). We next monitored different time points postinfection and found that *Zdhhc7^−/−^* BMDMs exhibited a strong reduction in pIRF3 at 6 h compared to WT (Fig. 3*B*). After 16 h infection, *Zdhhc7^−/−^* BMDMs still maintained a weak signal of pIRF3, while in WT BMDMs, pIRF3 was back to resting state due to the clearance of the virus (Fig. 3*B*).

Viral proteins are found to be palmitoylated by host ZDHHCs; for example, VSV G protein is palmitoylated by ZDHHC3/7 (24). To exclude the possibility that the reduced antiviral response in *Zdhhc7^−/−^* BMDMs was caused by palmitoylation-associated reduction of virus propagation, we assessed the virus level by blotting the IAV hemagglutinin (HA). Comparing to WT BMDMs, *Zdhhc7^−/−^* BMDMs contained more cellular IAV HA at both 6 and 16 h postinfection, and WT BMDMs essentially cleared the virus 16 h postinfection (Fig. 3*B*). For the induction of downstream antiviral genes, *Zdhhc7^−/−^* BMDMs induced less *Ifnb*, *Ifit1*, *Il6,* and *Rig-1* 8 h post-IAV infection (Fig. 3*C*), consistent with the less activated IRF3. Infecting BMDMs with another RNA virus, Encephalomyocarditis virus (EMCV), produced similar results, with *Zdhhc7^−/−^* BMDMs exhibiting reduced induction of type I interferon and interferon-stimulated genes (ISGs) at different time points and higher viral mRNA level compared to WT BMDMs (Fig. 3 *D* and *E*). Deletion of *Zdhhc7* in BMDMs also diminished NF-κB activation upon EMCV infection as evidenced by the decreased phosphorylation of NF-κB (*SI Appendix*, Fig. S5*L*).

Additionally, we studied the effect of 2-BP in the RLR signaling and antiviral response since it inhibited MAVS palmitoylation. 2-BP inhibited the induction of *IFNB* and *IL8* in poly(I:C) stimulated HEK293T cells (*SI Appendix*, Fig. S5*M*). In BMDMs, treatment of 2-BP significantly decreased the activation of IRF3 and the induction of *Ifnb* with poly(I:C) (Fig. 3 *F* and *G*). When infected by IAV, 2-BP inhibited pIRF3 in A549 cells (Fig. 3*H*) and suppressed induction of *IFNL2/3* and *IFNB* in both A549 and HEK293T cells (Fig. 3*I* and *SI Appendix*, Fig. S5 *N* and *O*). Taken together, our data support that ZDHHC7 significantly promotes RLR signaling and antiviral immune response.

### MAVS C508 Palmitoylation Is Important for RLR Signaling and Antiviral Immune Response.

**Fig. 4.**
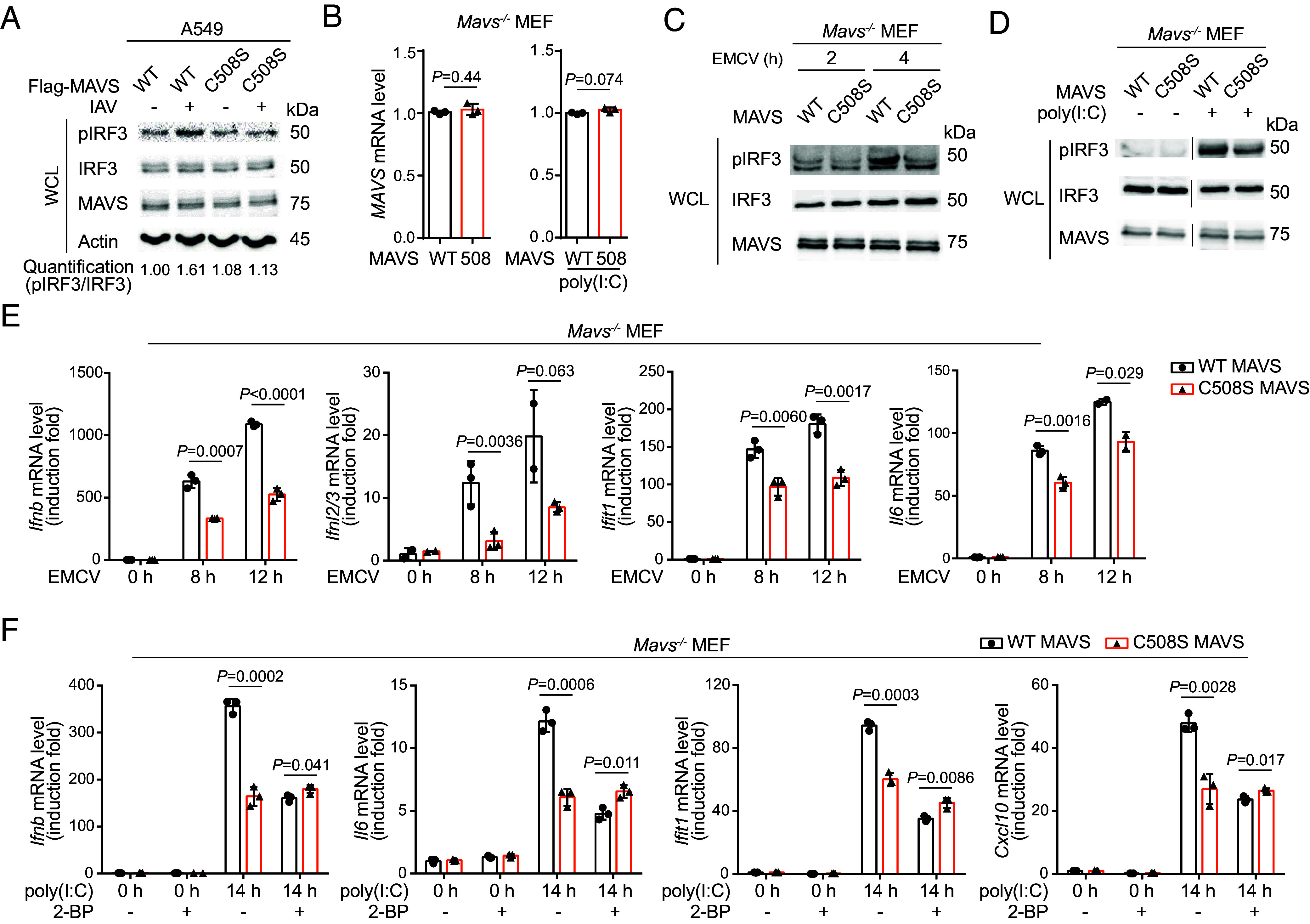
MAVS C508 palmitoylation is important for RLR signaling and antiviral immune response. (*A*) MAVS C508S mutation decreased IAV-induced IRF3 activation in A549 cells. A549 cells transfected with Flag-MAVS WT or C508S mutant for 24 h were infected with or without IAV for 8 h. Levels of pIRF3, IRF3, MAVS, and Actin were determined by immunoblots. The activation of IRF3 was quantified by pIRF3/IRF3. (*B*) Reintroduced MAVS WT or C508S were at similar mRNA levels. *Mavs^−/−^* MEF cells were stably transfected with WT or C508S MAVS and the mRNA levels at both resting and poly(I:C) stimulated states were analyzed using qPCR. (*C*, *D*) MAVS C508S mutation decreased IRF3 activation in MEF cells. *Mavs^−/−^* MEF cells stably expressing WT or C508S MAVS were infected with EMCV (*C*) or were stimulated by poly(I:C) transfection (*D*). Levels of pIRF3, IRF3, and MAVS were detected by immunoblots. (*E*) MAVS C508S mutation decreased EMCV-induced antiviral gene expression in MEF cells. *Mavs^−/−^* MEF cells stably expressing WT or C508S MAVS were infected by EMCV, and the inductions of *Ifnb*, *Ifnl2/3*, *Ifit1*, and *Il6* were detected using qPCR. (*F*) 2-BP treatment inhibited antiviral gene induction with WT MAVS but had little effect with MAVS C508 mutant. *Mavs^−/−^* MEF cells stably expressing WT or C508S MAVS were pretreated with 50 μM 2-BP or DMSO for 2 h and stimulated with or without poly(I:C) at 200 ng/mL for 14 h. Inductions of *Ifnb*, *Il6*, *Ifit1*, and *Cxcl10* were determined using qPCR. mRNA level in each sample was normalized to internal control (*Actb*). *P* values were calculated using two-tailed unpaired *t* tests. Data are mean ± SD.

Our data above showed that ZDHHC7-catalyzed palmitoylation promotes RLR signaling and antiviral immune response, but whether MAVS palmitoylation on C508 contributes to this activation still needs to be established. We thus evaluated the difference in IRF3 activation upon IAV infection in the presence of WT or C508S mutant MAVS. A549 cells transfected with WT Flag-MAVS had an elevated IRF3 phosphorylation level upon IAV infection, compared to cells transfected with the C508S mutant of Flag-MAVS (Fig. 4*A*). To eliminate the influence of endogenous MAVS, we generated *Mavs^−/−^* MEF cells stably expressing WT or C508S mutant MAVS at similar mRNA levels at both resting and RLR activated states (Fig. 4*B*). The C508S MAVS cells showed weaker pIRF3 signals with both EMCV infection and poly(I:C) stimulation compared to WT (Fig. 4 *C*, *D*). Consistent with this, decreased induction of *Ifnb*, *Ifnl2/3*, *Ifit1*, and *Il6* was observed in C508S MAVS MEF cells when infected with EMCV (Fig. 4*E*).

To further confirm that the effect caused by C508S mutant is dependent on ZDHHC-catalyzed palmitoylation but not potential structural change or other cysteine modifications, we treated the poly(I:C)-stimulated *Mavs^−/−^* MEFs expressing WT or C508S mutant MAVS with or without 2-BP. C508S MAVS had a weaker capability in the induction of *Ifnb*, *Il6*, *Ifit1*, and *Cxcl10* upon poly(I:C) transfection compared to WT MAVS. 2-BP treatment inhibited the induction of these genes in WT MEF cells but had little effect on the C508S mutant (Fig. 4*F*), indicating that the effect of C508S is largely through ZDHHC-catalyzed MAVS C508 palmitoylation. Collectively, the results suggest that MAVS C508 palmitoylation by ZDHHC7 is important for RLR signaling and antiviral immune response.

### ZDHHC7 Promotes Antiviral Innate Immunity In Vivo.

**Fig. 5.**
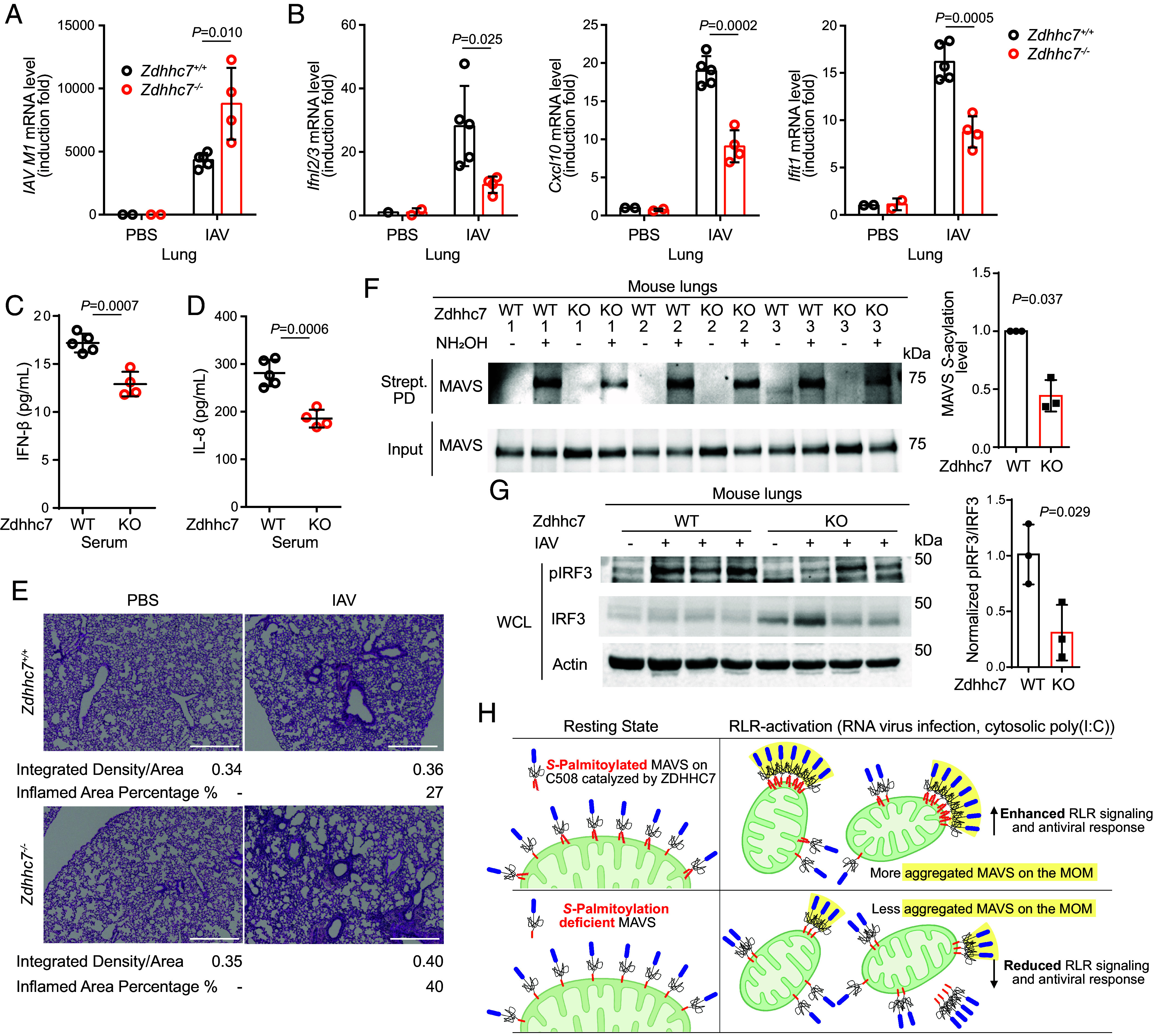
ZDHHC7 facilitates antiviral immune response in vivo. (*A* and *B*) *Zdhhc7^−/−^* mice had lower antiviral immune response compared to WT. WT and *Zdhhc7^−/−^* mice were intranasally infected with 1,000 pfu of IAV H1N1 strain A/PR/8/34 for 48 h. qPCR was used to measure IAV *M1* mRNA level (*A*) and induction of *Ifnl2/3*, *Cxcl10*, and *Ifit1* (*B*) in the mouse lungs. (*C* and *D*) ELISA analysis of IFN-β (*C*) and IL-8 (d) in serum from WT and *Zdhhc7^−/−^* mice infected by 1,000 pfu IAV for 48 h. (*E*) Representative H&E staining of the lung sections of PBS-treated or 48 h IAV-infected WT and *Zdhhc7^−/−^* mice (Scale bars, 500 μm.) The ratio of integrated density to total area and the percentage of inflamed area were quantified for each image. (*F*) ABE assay results showing the decreased MAVS *S*-palmitoylation levels in the lungs of *Zdhhc7^−/−^* mice infected by IAV compared to WT mice. Quantification of the results is shown on the right. (*G*) IAV-infected *Zdhhc7^−/−^* mouse lungs exhibited reduced IRF3 activation. Levels of pIRF3, IRF3, and Actin were detected by immunoblots. The ratio of pIRF3/ IRF3 was quantified and shown on the right. (*H*) Model of the regulation of MAVS by ZDHHC7-catalyzed C508 palmitoylation. At resting state, MAVS is anchored to the MOM via its C-terminal tail-anchor, while C508 palmitoylation does not affect its localization on the MOM. However, when the RLR signaling is activated by viral RNA or cytosolic poly(I:C), C508 palmitoylation by ZDHHC7 stabilizes and promotes MAVS aggregation on the MOM, enhancing the antiviral immune response. The illustration was created with BioRender.com. The mRNA level in each sample was normalized to internal control (*Actb*) (*A* and *B*). Number of WT mouse, n = 5; number of *Zdhhc7^−/−^* mouse, n = 4 (*A*–*D*). *P* values were calculated using two-tailed unpaired *t* tests (*A*–*D* and *G*) or two-tailed ratio paired *t* tests (*F*). Data are mean ± SD in *A*–*D* and *F*.

ZDHHC7 enhances antiviral response by promoting the gene expression of type I and type III interferons, and ISGs in cultured cells, and therefore, we hypothesized that ZDHHC7 is also important for antiviral innate immunity in vivo. We infected WT and *Zdhhc7^−/−^* mice with IAV H1N1 PR8 via intranasal administration. Forty-eight hours postinfection, higher IAV *M1* mRNA levels were detected in the lung of *Zdhhc7^−/−^* mice compared to the lung of WT animals, showing that ZDHHC7 deficient mice had higher IAV loads in the lung (Fig. 5*A* and *SI Appendix*, Fig. S6*A*). Upon IAV infection, the inductions of *Ifnl2/3*, *Cxcl10*, and *Ifit1* in the lungs were greater in WT mice than in *Zdhhc7^−/−^* mice (Fig. 5*B* and *SI Appendix*, Fig. S6*B*). WT mice showed higher levels of serum type I interferon IFN-β and cytokine IL-8 (Fig. 5 *C* and *D*). Furthermore, Hematoxylin and Eosin (H&E) staining of the lung sections of IAV-infected WT mice demonstrated less immune cell infiltration compared to the lungs of infected *Zdhhc7^−/−^* mice (Fig. 5*E* and *SI Appendix*, Fig. S6*C*). These results suggest that ZDHHC7 positively regulates antiviral innate immunity in vivo and restrains propagation of virus in the host. Consistent with the cellular data, *Zdhhc7^−/−^* mice showed lower MAVS *S*-acylation levels in the infected lungs compared to WT as examined by ABE assay (Fig. 5*F*), and the activation of IRF3 was lower in the *Zdhhc7^−/−^* infected mouse lung (Fig. 5*G*), suggesting that ZDHHC7-catalyzed MAVS C508 palmitoylation underlies the role of ZDHHC7 in antiviral immune response (Fig. 5*H*).

## Discussion

As a key adaptor protein in the RLR signaling pathway, MAVS forms unique prion-like aggregation that serves as a platform for efficient signaling propagation upon virus infection (7, 10). The aggregation and activation of MAVS are tightly regulated by its interacting proteins, isoforms, localization, and posttranslational modifications (15–17, 19). MAVS C508 has been previously identified as a cleavage site of hepatitis C virus protease NS3/4A (41), and here, we identify another important regulatory axis at this residue–*S*-palmitoylation regulated by ZDHHC7. Inhibition of MAVS C508 palmitoylation by ZDHHC7 deletion or C508S mutation did not affect the mitochondrial localization of MAVS at resting state but reduced its aggregation and mitochondrial localization upon RLR activation and, consequently, down-regulated the production of type I and III interferons and ISGs. Our findings demonstrate the important role of ZDHHC7 and protein palmitoylation in regulating antiviral immune response both in cells and in vivo.

The localization of tail-anchored proteins to different membranes (e.g., ER, mitochondrial outer membrane, and peroxisomal membrane) is determined by their C-terminal single transmembrane domain with the facilitation of molecular chaperones and receptors such as mitochondrial import receptors (TOMs) and peroxisomal biogenesis factors (PEXs) (42). Therefore, unlike the translocation of cytosolic proteins to membrane by lipidation (e.g., prenylation and *S*-palmitoylation of RAS GTPases) (43, 44), tail-anchored proteins or other integral membrane proteins typically do not require lipidation for membrane targeting. However, many tail-anchored proteins and integral membrane proteins are indeed *S*-palmitoylated (45, 46). A theory that has been mentioned in many studies is that *S*-palmitoylation promotes lipid raft targeting (47). Lipid raft is a relatively controversial concept that essentially represents lipid phase separation in the membrane bilayer (48). We propose another possibility that *S*-palmitoylation does not promote lipid raft targeting, but instead, promotes the phase separation of membrane proteins. In other words, the previously described function of *S*-palmitoylation in promoting lipid raft localization of proteins is more likely promoting protein phase separation on the membrane. So “lipid raft” is more a protein phase separation than a lipid phase separation, although some rearrangement of the lipid molecules may accompany the protein phase separation. MAVS *S*-palmitoylation at C508 is an example supporting this hypothesis since the mitochondria typically are considered not to have “lipid rafts”(49, 50). Notably, MAVS bears a huge intrinsically disordered region. The presence of phase-separated MAVS aggregates (20) might drive the bending of the membrane of mitochondria (51), as observed in the SR-SIM images of A549 and BMDMs that MAVS showed a curved distribution (Fig. 1*I* and *SI Appendix*, Fig. S3*F*). An enhanced protein affinity to membrane conferred by *S*-palmitoylation might be important for maintaining membrane binding under high membrane curvature since palmitoyl chain is known to demonstrate high-curvature selectivity (52). Similarly, caveolin, a marker of caveolae, is able to induce membrane curvature, and palmitoylation of caveolin, occurring close to its hydrophobic domain, is important for its “lipid raft” localization (i.e., phase condensation) (53). Moreover, protein palmitoylation is known to be important for concentrating cargo proteins in the most highly curved regions of the Golgi membranes to help protein sorting and trafficking (24). In these cases, the role of *S*-palmitoylation is again to help maintain the membrane binding in the highly curved membrane regions.

MAVS, when activated, forms filaments approximately 400 nm in length containing hundreds of MAVS molecules (12), which is observed in this study using SR-SIM in A549, BMDM, and MEF cells with virus infection (Fig. 1*I* and *SI Appendix*, Fig. S3*F*) or poly(I:C) transfection (Fig. 2*I* and *SI Appendix*, Fig. S4*H*). Also, infection or poly(I:C) transfection induces mitochondrial fragmentation, as observed in the current and previous studies (Fig. 1*H*) (54). It is possible that the mitochondrial outer membrane curvature change resulting from MAVS aggregation can further impact mitochondrial function, such as promoting mitochondria fission and loss of mitochondrial membrane potential, which both up-regulate mitochondrial reactive oxygen species. Palmitoylation might further promote these processes, as in ZDHHC7 WT cells the hyperaggregated MAVS aligned with less healthy mitochondria and loss of mitochondrial membrane potential (*SI Appendix*, Fig. S3*F* and S4*H*). Whether and how palmitoylation of MAVS by ZDHHC7 directly regulates mitochondrial metabolism and function upon virus infection awaits further investigation.

Upregulation of mitochondrial reactive oxygen species has been associated with enhanced aggregation and activation of MAVS as well as increased expression of type I interferon, which is one of the causes of autoimmune diseases such as systemic lupus erythematosus (55). MAVS C79F mutation, which resides close to the CARD that is crucial for the aggregation, relieves the lupus symptoms of patients due to the reduced MAVS aggregation (39). Since we revealed that ZDHHC7 increases MAVS oligomerization and enhances type I interferon production, inhibition of ZDHHC7 might be a therapeutic strategy alleviating these autoimmune diseases. Indeed, ZDHHC7 has been shown to be involved in several immune pathways, including cGAS-STING, NLPR3 inflammasome, and JAK-STAT pathways, (27, 29–31) and ZDHHC7 is highly expressed in immune cells such as monocytes and macrophages (30). These studies highlight ZDHHC7 as a key regulator in multiple immune responses and suggest that targeting ZDHHC7 is a promising strategy to tune the immune responses to treat inflammatory and autoimmune diseases.

## Methods

### Mice.

The *Zdhhc7* knockout mouse strain B6.129P2(FVB)-Zdhhc7tm1.2Lusc/Mmmh, RRID: MMRRC_043511-MU was obtained from the Mutant Mouse Resource and Research Center (MMRRC) at the University of Missouri (29). The *Zdhhc3* knockout mice were generated by CRISPR/Cas9 technique (30). Mice were housed in a specific pathogen-free facility. The mouse protocols were approved by Cornell University’s Institutional Animal Care and Use Committee and were strictly followed when performing experiments.

### Antibodies, Reagents, and Plasmids.

Antibodies from commercial sources include MAVS (24930, CST, 1:1000 dilution), MAVS (sc-166583, Santa Cruz, 1:100 dilution for immunofluorescence), Flag HRP (A8592, Millipore, 1:5000 dilution), IRF3 (4302, CST, 1:1000 dilution), phospho-IRF-3 (Ser396) (4947, CST, 1:1000 dilution), β-Actin (C4) (sc-47778, Santa Cruz, 1:1000 dilution), HA-Tag (F-7) (sc-7392, Santa Cruz, 1:1000 dilution), Calnexin (2433, CST, 1:1000 dilution), VDAC (4866, CST, 1:1000 dilution), anti-mouse IgG HRP (7076S, CST, 1:2500 dilution), anti-rabbit IgG HRP (7074S, CST, 1:2500 dilution), ZDHHC7 (R12-3691, Assay Biotechnology, 1:100 dilution), and Alexa Fluor 488 Goat anti-Rabbit IgG (H + L) (A-11008, Invitrogen). Flag-MAVS and 3xFlag-miniMAVS plasmids were obtained from Addgene. The plasmid encoding untagged MAVS with a CMV promoter for lentivirus preparation was obtained from GeneCopoeia (EX-Z0814-Lv105). Murine ZDHHC1-23, PPTs, and ABHDs plasmids were kindly provided by Dr. Masaki Fukata. All the MAVS cysteine mutations were generated by site-directed mutagenesis. ZDHHC7 siRNA (sc-93249, Santa Cruz).

### Chemical and Biochemical Reagents.

Chemical and biochemical reagents used included tetramethylrhodamine-azide (TAMRA-azide, 7130, Lumiprobe), tris[(1-benzyl-1H-1,2,3-triazol-4-yl)methyl]amine (TBTA, T2993, TCI Chemicals), CuSO_4_ (TCI Chemicals), tris(2-carboxyethyl)phosphine hydrochloride (TCEP, C4706, Sigma-Aldrich), 2-bromopalmitic acid (2-BP, 21604, Sigma-Aldrich), polyethylenimine hydrochloride (PEI, Polysciences), poly(I:C) HMW (tlrl-pic, InvivoGen), protease inhibitor cocktail (P8340, Sigma-Aldrich), phosphatase inhibitor cocktail (P0044, Sigma), universal nuclease (88700, Thermo Fisher), dithiothreitol (DTT100, Goldbio), Pierce ECL Western Blotting Substrate (Thermo Fisher), clarity max western ECL substrate (1705062, Bio-Rad), high-capacity streptavidin agarose (20359, Thermo Fisher), anti-Flag agarose gel (A2220, Sigma-Aldrich), hydroxylamine (50% solution in water, Sigma-Aldrich), and N-ethylmaleimide (NEM, Sigma-Aldrich). The IFN beta Mouse ELISA Kit was purchased from Invitrogen (424001), and the Mouse IL-8 ELISA Kit was from MyBioSource (MBS7606860). 15-Hexadecynoic acid (Alk14) was synthesized in house.

### Cells and Viruses.

HEK293T cells from ATCC were cultured using Dulbecco’s modified Eagle’s medium (DMEM, Gibco) supplemented with heat-inactivated 10% calf serum (CS, Sigma C8056). *ZDHHC7^−/−^* HEK293T cells were generated by CRISPR/Cas9 technique (29) and had been validated via immunoblotting before use. Mouse BMDMs were isolated from the tibia and femur of 8-12 wk mice as previously described (56). BMDMs were cultured in DMEM supplemented with 20% heat-inactivated fetal bovine serum (FBS, Gibco), 20 ng/mL Macrophage Colony-Stimulating Factor human (M-CSF, Sigma-Aldrich), and 1× Antibiotic-Antimycotic (Gibco) for 5 d at 37 °C before use. A549 cells were grown in RPMI-1640 (11875085, Thermo Fisher) with 10% heat-inactivated FBS. *Mavs^−/−^* MEF cells were provided by Dr. Zhijian (James) Chen (33). *Mavs^−/−^* MEF cells stably expressing WT or C508S mutant MAVS were generated by lentivirus transduction. Primary *Zdhhc7^+/+^* and *Zdhhc7^−/−^* MEF cells were isolated from *Zdhhc7^+/-^* embryos at day 12 and validated at both the genome DNA and the mRNA level.

Influenza A virus (H1N1) strain A/PR/8/34 and EMCV were obtained from ATCC (VR-95, VR-129B). IAV was propagated in Vero cells (ATCC CCL-81) cultured in Eagle’s Minimum Essential Medium (EMEM, 30-2003, ATCC) with 0.3% Bovine Albumin Fraction V (BSA, Thermo Fisher), and 1 μg/mL TPCK-treated trypsin, as previously described (56). EMCV was propagated in Vero cells cultured in EMEM with 0.3% BSA. Virus-containing supernatant was collected when cells exhibited 80% cytopathic effects.

### Detection of MAVS *S*-Palmitoylation by In-Gel Fluorescence.

HEK293T cells were transfected with Flag-MAVS and ZDHHC7-HA using PEI for 24 h and were treated with 50 μM Alk14 for 6 h before collecting. Cells were washed and lysed in 4% SDS lysis buffer (50 mM triethanolamine, 150 mM NaCl, 4% (w/v) SDS, protease inhibitor cocktail 1:100 dilution, and nuclease 1:1000 dilution, pH 7.4). The lysate was diluted to 1% SDS with 1% NP-40 lysis buffer (25 mM Tris-HCl pH 8.0, 10% glycerol, 150 mM NaCl, and 1% NP-40). Flag-MAVS was purified by anti-Flag agarose beads, and the click chemistry mixture consisting of 40 μL IP wash buffer (25 mM Tris-HCl pH 8.0, 150 mM NaCl, and 0.2% NP-40), 2 μL of 2 mM TAMRA-azide, 2.4 μL of 10 mM TBTA, 2 μL of 40 mM CuSO_4_, and 2 μL of 40 mM TCEP was added to each sample. The mixture was vortexed and incubated in the dark at room temperature for 30 min, followed by addition of 10 μL of 6× SDS loading dye. The samples were boiled at 95 °C for 10 min to denature proteins. Hydroxylamine was added to 0.4 M to cleave *S*-palmitoylation. Proteins were separated by SDS-PAGE, and the fluorescence signal was recorded by ChemiDoc Imaging Systems (Bio-Rad) using the Rhodamine channel. The gel was stained by Coomassie brilliant blue (B7920, Sigma) to demonstrate equal protein loading. Quantification of gel bands was performed using ImageJ.

### Acyl-Biotin Exchange Assay.

Cells cultured in a 10-cm dish were collected and lysed in 1.2 mL 2.5% SDS lysis buffer (100 mM Tris-HCl pH 7.2, 5 mM EDTA, 150 mM NaCl, 2.5% SDS, protease inhibitor cocktail 1:100 dilution, and nuclease (1:1000 dilution) containing additional 50 mM NEM). The lysate was incubated at room temperature for 2 h. The protein concentration was determined using a pierce BCA protein assay kit (23225, Thermo Fisher) and normalized. Protein was precipitated with cold methanol, chloroform, and water (4:1:3, v/v/v), air-dried, and redissolved by sonication in 1.2 mL of 2.5% SDS lysis buffer containing additional 5 mM biotin-HPDP (16459, Cayman Chemical). Samples were aliquoted into two and incubated with 0.5 mL of 1 M NH_2_OH or 1 M NaCl at room temperature for 2 h. Protein was precipitated and redissolved in 0.6 mL of 2.5% SDS lysis buffer containing 8 M Urea. Then, 40 µL of lysate was saved as input, and the rest was diluted with 14 mL PBS and subjected to streptavidin pull-down at room temperature for 2 h. Streptavidin beads were washed with PBS containing 1% SDS for 3 times. The beads were mixed with 2× SDS loading buffer and heated at 95 °C for 15 min. Samples were then resolved by SDS–PAGE for immunoblotting.

### Immunoprecipitation of Endogenous MAVS.

A total of 1 × 10^7^ THP-1 cells were seeded in 10 cm dish and differentiated with 10 ng/mL PMA overnight in RPMI 1640 media containing 10% FBS. Cells were harvested and lysed with RIPA buffer containing Protease Inhibitor Cocktail (P8849, Sigma) and nuclease (88702, Pierce) and then incubated on ice for 30 min. The lysate was centrifuged at 4 °C at 3,000 g for 5 min. Fifty microliters of supernatant was saved as input samples. A volume of 900 μL of supernatant was incubated with anti-MAVS or anti-IgG antibody (1:50) for 2 h at 4 °C. Fifty microliters of protein A/G conjugated magnetic beads (88802, Thermo Fisher) was added and incubated overnight for the immunoprecipitation of the endogenous MAVS proteins (IP samples). The beads were washed for three times; MAVS-interacting ZDHHC7 protein in the IP samples was accessed with immunoblotting analysis.

### Poly(I:C) Stimulation and Virus Infection In Vitro.

Poly(I:C) HMW was transfected with Lipofectamine RNAiMAX (13778150, Thermo Fisher) based on the manufacturer’s instructions for RLR stimulation or was directly added into the cell culture medium for TLR3 stimulation. Cells were seeded into 6-well plates 24 h before infection and were infected with IAV or EMCV at the indicated multiplicity of infection (MOI). Cell media were removed before virus inoculation. After incubation at 37 °C for 1 h, fresh medium was added. Cells were then lysed in 4% SDS lysis buffer for immunoblotting or subjected to RNA extraction for RT-qPCR.

### Real-Time Quantitative PCR.

Cells or tissue were extracted using the Total RNA Kit I (Omega Bio-tek). cDNA was obtained by the SuperScript VILO cDNA Synthesis Kit (Thermo Fisher) or High-Capacity cDNA Reverse Transcription Kit (Thermo Fisher). The qPCR was performed using SYBR Green PCR Master Mix (Thermo Fisher) or 2× Universal SYBR Green Fast qPCR Mix (Abclonal) with QuantStudio™ 7 Flex Real-Time PCR System. Relative expression of genes was calculated using the ΔΔCq method and normalized to *ACTB*. Primer sequences are listed in *SI Appendix*, Table S1.

### Western blot.

Whole-cell lysates were prepared in 4% SDS lysis buffer. Immunoblotting was performed using a standard protocol, and the signals developed with Pierce ECL Western Blotting Substrate (Thermo Fisher) or Clarity Max Western ECL Substrate (Bio-Rad) were recorded using Imaging Systems (Bio-Rad).

### Subcellular Fractionation.

Cells were homogenized in Buffer A (10 mM Tris-HCl at pH 7.5, 10 mM KCl, 1.5 mM MgCl_2_, 0.25 M D-mannitol, and protease inhibitor cocktail) by passing through plastic syringes (3-mL) fitted with a 25-gauge needle, and the lysate was centrifuged at 1,000 g for 10 min at 4 °C. The resulting supernatant (S1) was further centrifuged at 10,000g for 10 min at 4 °C, and supernatant 5 (S5) and crude mitochondria pellet 5 (P5) were obtained. P5 was solubilized in 2× SDS loading buffer for SDS-PAGE. Separation of the mitochondria-associated membrane and the mitochondria was performed as previously described (7).

### Immunofluorescence.

Cells were cultured in 35-mm glass bottom dishes (MatTek). Mitochondria were stained by MitoTracker Deep Red FM (Invitrogen) according to the manufacturer’s instructions. Cells were washed with PBS and fixed with 4% paraformaldehyde (PFA, v/v in PBS) for 20 min at room temperature. Cells were briefly air-dried after 4% PFA was removed, washed with PBS, permeabilized, and blocked with 5% BSA in PBS containing 0.1% saponin for 60 min at room temperature. The permeabilized cells were incubated with primary antibody overnight at 4 °C and then incubated with secondary antibody for 1 h at room temperature. Cells were washed with PBS containing 0.1% saponin three times before mounted in DAPI Fluoromount-G (0100-20, SouthernBiotech). The samples were imaged by an inverted confocal microscope (LSM710, Zeiss) or Elyra superresolution structured illumination microscope (SR-SIM) (Zeiss). The images were processed by Fiji ImageJ. Image quantification was performed using CellProfiler [v4.1.3 (1)] (57). MAVS clusters were segmented, and the size was measured using max ferret diameter. The overlaid histogram with fitted Gaussian distribution was plotted using python script based on python packages Matplotlib and SciPy. Comparison of the sizes of MAVS aggregates between WT and *Zdhhc7^−/−^* cells uses a cutoff at 100 nm to filter the nonoligomerized MAVS. The scatter plot with individual points was plotted using GraphPad Prism version 10.2.2.

### Semidenaturing Detergent Agarose Gel Electrophoresis (SDD-AGE).

Crude mitochondria P5 was solubilized in 1 × SDD loading dye (10% Glycerol, 0.5 × TBE, 2% SDS, and 0.0025% Bromophenol Blue) and was loaded onto a vertical 1.5% agarose gel. The electrophoresis was performed in 1 × TBE buffer containing 0.1% SDS for 40 min at 100Â V at 4 °C followed by transfer at 12 V for 15 h overnight at 4 °C.

### Virus Infection in vivo.

Intranasal IAV infection of mice was conducted as previously described (58). Age- and sex-matched *Zdhhc7* wild-type and knockout mice were infected with Influenza A virus (H1N1) strain A/PR/8/34 at 1,000 pfu/mouse. At 48 h postinfection, the mice were killed, and lungs and sera were collected. Lungs were homogenized in cold PBS at 1:1 ratio (buffer volume per tissue weight). Five percent of the lung homogenate was used for RNA extraction and RT-qPCR analysis of the host antiviral gene and IAV gene. Thirty percent of the lung homogenate was used for ABE assay, during which proteins were precipitated once before NEM blocking. IFN-β and IL-8 in the mouse serum were detected by the ELISA following the manufacturer’s instruction. For the evaluation of lung histology, lungs of IAV-infected (48 h postinfection) mice were collected, fixed with 4% PFA, and stained with hematoxylin and eosin (H&E). The H&E-stained images were visualized with Agilent BioTek Cytation 5 and quantified by ImageJ.

### Statistical analysis.

Statistical analyses were performed using GraphPad Prism 6. *P* values were calculated using two-tailed unpaired *t* tests unless otherwise noted.

## Supplementary Material

Appendix 01 (PDF)

## Data Availability

All study data are included in the article and/or supporting information.

## References

[1] M. Yoneyama , The RNA helicase RIG-I has an essential function in double-stranded RNA-induced innate antiviral responses. Nat. Immunol. **5**, 730–737 (2004). 15208624 10.1038/ni1087

[2] D. Kang , mda-5: An interferon-inducible putative RNA helicase with double-stranded RNA-dependent ATPase activity and melanoma growth-suppressive properties. Proc. Natl. Acad. Sci. U.S.A. **99**, 637–642 (2002). 11805321 10.1073/pnas.022637199 PMC117358

[3] R. B. Seth, L. Sun, C. K. Ea, Z. J. Chen, Identification and characterization of MAVS, a mitochondrial antiviral signaling protein that activates NF-κB and IRF3. Cell **122**, 669–682 (2005). 16125763 10.1016/j.cell.2005.08.012

[4] T. Kawai , IPS-1, an adaptor triggering RIG-I- and Mda5-mediated type I interferon induction. Nat. Immunol. **6**, 981–988 (2005). 16127453 10.1038/ni1243

[5] E. Meylan , Cardif is an adaptor protein in the RIG-I antiviral pathway and is targeted by hepatitis C virus. Nature **437**, 1167–1172 (2005). 16177806 10.1038/nature04193

[6] L.-G. Xu , VISA is an adapter protein required for virus-triggered IFN-β signaling. Mol. Cell **19**, 727–740 (2005). 16153868 10.1016/j.molcel.2005.08.014

[7] F. Hou , MAVS forms functional prion-like aggregates to activate and propagate antiviral innate immune response. Cell **146**, 448–461 (2011). 21782231 10.1016/j.cell.2011.06.041 PMC3179916

[8] S. Liu Phosphorylation of innate immune adaptor proteins MAVS, STING, and TRIF induces IRF3 activation. Science **347**, aaa2630 (2015). 25636800 10.1126/science.aaa2630

[9] T. Kawai, S. Akira, The role of pattern-recognition receptors in innate immunity: Update on Toll-like receptors. Nat. Immunol. **11**, 373–384 (2010). 20404851 10.1038/ni.1863

[10] X. Cai, Z. J. Chen, Prion-like polymerization as a signaling mechanism. Trends Immunol. **35**, 622–630 (2014). 25457352 10.1016/j.it.2014.10.003 PMC4429004

[11] S. Ding, M. D. Robek, Peroxisomal MAVS activates IRF1-mediated IFN-λ production. Nat. Immunol. **15**, 700–701 (2014). 25045870 10.1038/ni.2924

[12] H. Xu , Structural basis for the prion-like MAVS filaments in antiviral innate immunity. Elife **3**, e01489 (2014). 24569476 10.7554/eLife.01489 PMC3932521

[13] R. Fang , MAVS activates TBK1 and IKKε through TRAFs in NEMO dependent and independent manner. PLoS Pathog. **13**, e1006720 (2017). 29125880 10.1371/journal.ppat.1006720 PMC5699845

[14] S. W. Brubaker, A. E. Gauthier, E. W. Mills, N. T. Ingolia, J. C. Kagan, A bicistronic MAVS transcript highlights a class of truncated variants in antiviral immunity. Cell **156**, 800–811 (2014). 24529381 10.1016/j.cell.2014.01.021 PMC3959641

[15] F. You , PCBP2 mediates degradation of the adaptor MAVS via the HECT ubiquitin ligase AIP4. Nat. Immunol. **10**, 1300–1308 (2009). 19881509 10.1038/ni.1815

[16] J. Li , WDR77 inhibits prion-like aggregation of MAVS to limit antiviral innate immune response. Nat. Commun. **14**, 4824 (2023). 37563140 10.1038/s41467-023-40567-5 PMC10415273

[17] N. Qi , Multiple truncated isoforms of MAVS prevent its spontaneous aggregation in antiviral innate immune signalling. Nat. Commun. **8**, 15676 (2017). 28607490 10.1038/ncomms15676 PMC5474743

[18] S. Z. Li , Phosphorylation of MAVS/VISA by Nemo-like kinase (NLK) for degradation regulates the antiviral innate immune response. Nat. Commun. **10**, 1–14 (2019). 31324787 10.1038/s41467-019-11258-x PMC6642205

[19] B. Liu, C. Gao, Regulation of MAVS activation through post-translational modifications. Curr. Opin. Immunol. **50**, 75–81 (2018). 29245018 10.1016/j.coi.2017.12.002

[20] T. Dai , MAVS deSUMOylation by SENP1 inhibits its aggregation and antagonizes IRF3 activation. Nat. Struct. Mol. Biol. **30**, 785–799 (2023). 37188808 10.1038/s41594-023-00988-8

[21] J. Zhu , Arginine monomethylation by PRMT7 controls MAVS-mediated antiviral innate immunity. Mol. Cell **81**, 3171–3186.e8 (2021). 34171297 10.1016/j.molcel.2021.06.004

[22] X. Bai , The protein arginine methyltransferase PRMT9 attenuates MAVS activation through arginine methylation. Nat. Commun. **13**, 5016 (2022). 36028484 10.1038/s41467-022-32628-y PMC9418238

[23] H. Jiang , Protein lipidation: Occurrence, mechanisms, biological functions, and enabling technologies. Chem. Rev. **118**, 919–988 (2018). 29292991 10.1021/acs.chemrev.6b00750 PMC5985209

[24] A. M. Ernst , S-palmitoylation sorts membrane cargo for anterograde transport in the golgi. Dev. Cell **47**, 479–493.e7 (2018). 30458139 10.1016/j.devcel.2018.10.024 PMC6251505

[25] Z. Wu , Palmitoylation of SARS-CoV-2 S protein is essential for viral infectivity. Signal Transduct. Target Ther. **6**, 231 (2021). 34117209 10.1038/s41392-021-00651-y PMC8193602

[26] H. Lin, Protein cysteine palmitoylation in immunity and inflammation. FEBS J. **288**, 7043–7059 (2021). 33506611 10.1111/febs.15728 PMC8872633

[27] K. Mukai , Activation of STING requires palmitoylation at the Golgi. Nat. Commun. **7**, 11932 (2016). 27324217 10.1038/ncomms11932 PMC4919521

[28] Y. Lu , Palmitoylation of NOD1 and NOD2 is required for bacterial sensing. Science **1979**, 460–467 (2019). 10.1126/science.aau6391 31649195

[29] M. Zhang , A STAT3 palmitoylation cycle promotes TH17 differentiation and colitis. Nature **586**, 434–439 (2020). 33029007 10.1038/s41586-020-2799-2 PMC7874492

[30] T. Yu NLRP3 Cys126 palmitoylation by ZDHHC7 Promotes inflammasome activation. Cell Rep. **43**, 114070 (2024), 10.1101/2023.11.07.566005. 38583156 PMC11130711

[31] L. Wang , Palmitoylation prevents sustained inflammation by limiting NLRP3 inflammasome activation through chaperone-mediated autophagy. Mol. Cell **83**, 281–297.e10 (2023). 36586411 10.1016/j.molcel.2022.12.002

[32] Y.-C. Kim , Toll-like receptor mediated inflammation requires FASN-dependent MYD88 palmitoylation. Nat. Chem. Biol. **15**, 907–916 (2019). 31427815 10.1038/s41589-019-0344-0

[33] Q. Sun , The specific and essential role of MAVS in antiviral innate immune responses. Immunity **24**, 633–642 (2006). 16713980 10.1016/j.immuni.2006.04.004

[34] T. Lan, C. Delalande, B. C. Dickinson, Inhibitors of DHHC family proteins. Curr. Opin. Chem. Biol. **65**, 118–125 (2021). 34467875 10.1016/j.cbpa.2021.07.002 PMC8671176

[35] E. L. Huttlin , Dual proteome-scale networks reveal cell-specific remodeling of the human interactome. Cell **184**, 3022–3040.e28 (2021). 33961781 10.1016/j.cell.2021.04.011 PMC8165030

[36] T. M. Maynard , Mitochondrial localization and function of a subset of 22q11 deletion syndrome candidate genes. Mol. Cellular Neurosci. **39**, 439–451 (2008). 10.1016/j.mcn.2008.07.027 PMC2729512 18775783

[37] D. Davda, B. R. Martin, Acyl protein thioesterase inhibitors as probes of dynamic *S*-palmitoylation. Medchemcomm **5**, 268–276 (2014). 25558349 10.1039/C3MD00333G PMC4280026

[38] S. M. Horner, H. M. Liu, H. S. Park, J. Briley, M. Gale, Mitochondrial-associated endoplasmic reticulum membranes (MAM) form innate immune synapses and are targeted by hepatitis C virus. Proc. Natl. Acad. Sci. U.S.A. **108**, 14590–14595 (2011). 21844353 10.1073/pnas.1110133108 PMC3167523

[39] I. A. Buskiewicz , Reactive oxygen species induce virus-independent MAVS oligomerization in systemic lupus erythematosus. Sci. Signal **9**, ra115 (2016). 27899525 10.1126/scisignal.aaf1933 PMC5321043

[40] L. de B. Monteiro, G. G. Davanzo, C. de F. Aguiar, P. M. M. Moraes-Vieira, Using flow cytometry for mitochondrial assays. MethodsX **7**, 100938 (2020). 32551241 10.1016/j.mex.2020.100938 PMC7289760

[41] X.-D. Li, L. Sun, R. B. Seth, G. Pineda, Z. J. Chen, Hepatitis C virus protease NS3/4A cleaves mitochondrial antiviral signaling protein off the mitochondria to evade innate immunity. Proc. Natl. Acad. Sci. U.S.A. **102**, 17717–17722 (2005). 16301520 10.1073/pnas.0508531102 PMC1308909

[42] N. Borgese, S. Brambillasca, S. Colombo, How tails guide tail-anchored proteins to their destinations. Curr. Opin. Cell Biol. **19**, 368–375 (2007). 17629691 10.1016/j.ceb.2007.04.019

[43] J. Gao, J. Liao, G.-Y. Yang, CAAX-box protein, prenylation process and carcinogenesis. Am. J. Transl. Res. **1**, 312–25 (2009). 19956441 PMC2776320

[44] S. Eisenberg , The role of palmitoylation in regulating Ras localization and function. Biochem. Soc. Trans. **41**, 79–83 (2013). 23356262 10.1042/BST20120268

[45] S. Blaskovic, M. Blanc, F. G. van der Goot, What does *S*-palmitoylation do to membrane proteins? FEBS J. **280**, 2766–2774 (2013). 23551889 10.1111/febs.12263

[46] B. Zhang , Amino acid and protein specificity of protein fatty acylation in *Caenorhabditis elegans*. Proc. Natl. Acad. Sci. U.S.A. **121** (2024). 10.1073/pnas.2307515121 PMC10835129 38252833

[47] I. Levental, D. Lingwood, M. Grzybek, Ü. Coskun, K. Simons, Palmitoylation regulates raft affinity for the majority of integral raft proteins. Proc. Natl. Acad. Sci. U.S.A. **107**, 22050–22054 (2010). 21131568 10.1073/pnas.1016184107 PMC3009825

[48] D. Lingwood, K. Simons, Lipid rafts as a membrane-organizing principle. Science **1979**, 46–50 (2010). 10.1126/science.1174621 20044567

[49] T. Bae , Lipid raft proteome reveals ATP synthase complex in the cell surface. Proteomics **4**, 3536–3548 (2004). 15378739 10.1002/pmic.200400952

[50] Y. Z. Zheng, K. B. Berg, L. J. Foster, Mitochondria do not contain lipid rafts, and lipid rafts do not contain mitochondrial proteins. J. Lipid Res. **50**, 988–998 (2009). 19136664 10.1194/jlr.M800658-JLR200 PMC2666185

[51] A. Mahapatra, D. Saintillan, P. Rangamani, Curvature-driven feedback on aggregation-diffusion of proteins in lipid bilayers. Soft Matter **17**, 8373–8386 (2021). 34550131 10.1039/d1sm00502b PMC8462121

[52] N. S. Hatzakis , How curved membranes recruit amphipathic helices and protein anchoring motifs. Nat. Chem. Biol. **5**, 835–841 (2009). 19749743 10.1038/nchembio.213

[53] A. Uittenbogaard, E. J. Smart, Palmitoylation of caveolin-1 is required for cholesterol binding, chaperone complex formation, and rapid transport of cholesterol to caveolae. J. Biol. Chem. **275**, 25595–25599 (2000). 10833523 10.1074/jbc.M003401200

[54] L. Zhang, Y. Qin, M. Chen, Viral strategies for triggering and manipulating mitophagy. Autophagy **14**, 1665–1673 (2018). 29895192 10.1080/15548627.2018.1466014 PMC6135629

[55] K. B. Elkon, V. V. Stone, Type I interferon and systemic lupus erythematosus. J. Interferon. Cytokine Res. **31**, 803–812 (2011). 21859344 10.1089/jir.2011.0045 PMC3216059

[56] G. Toda, T. Yamauchi, T. Kadowaki, K. Ueki, Preparation and culture of bone marrow-derived macrophages from mice for functional analysis. STAR Protoc. **2**, 100246 (2021). 33458708 10.1016/j.xpro.2020.100246 PMC7797923

[57] D. R. Stirling , Cell Profiler 4: Improvements in speed, utility and usability. BMC Bioinform. **22**, 433 (2021). 10.1186/s12859-021-04344-9 PMC8431850 34507520

[58] I.-E. Galani, V. Triantafyllia, E.-E. Eleminiadou, E. Andreakos, Protocol for influenza A virus infection of mice and viral load determination. STAR Protoc. **3**, 101151 (2022). 35146450 10.1016/j.xpro.2022.101151 PMC8819391

