# Supplementary material for: MAVS Cys508 palmitoylation promotes its aggregation on the mitochondrial outer membrane and antiviral innate immunity
Source: Proc Natl Acad Sci U S A. 2024 Aug 14;121(34):e2403392121. doi: 10.1073/pnas.2403392121 (PMC11348129; doi:10.1073/pnas.2403392121)
Supplement: Supplementary file 1 — Appendix 01 (PDF) [file pnas.2403392121.sapp.pdf]

Supporting Information for

MAVS Cys508 palmitoylation promotes its aggregation on the mitochondrial outer membrane and antiviral innate immunity

Yinong Liu[a], Dan Hou[a], Wenzhe Chen[a], Xuan Lu[a], Garrison P. Komaniecki[a,1], Yilai Xu[a], Tao Yu[a], Sophia M. Zhang[a], Maurine E. Linder[b], Hening Lin[a,c,2]

Hening Lin
Email: hl379@cornell.edu

This PDF file includes:

Figures S1 to S6
Table S1

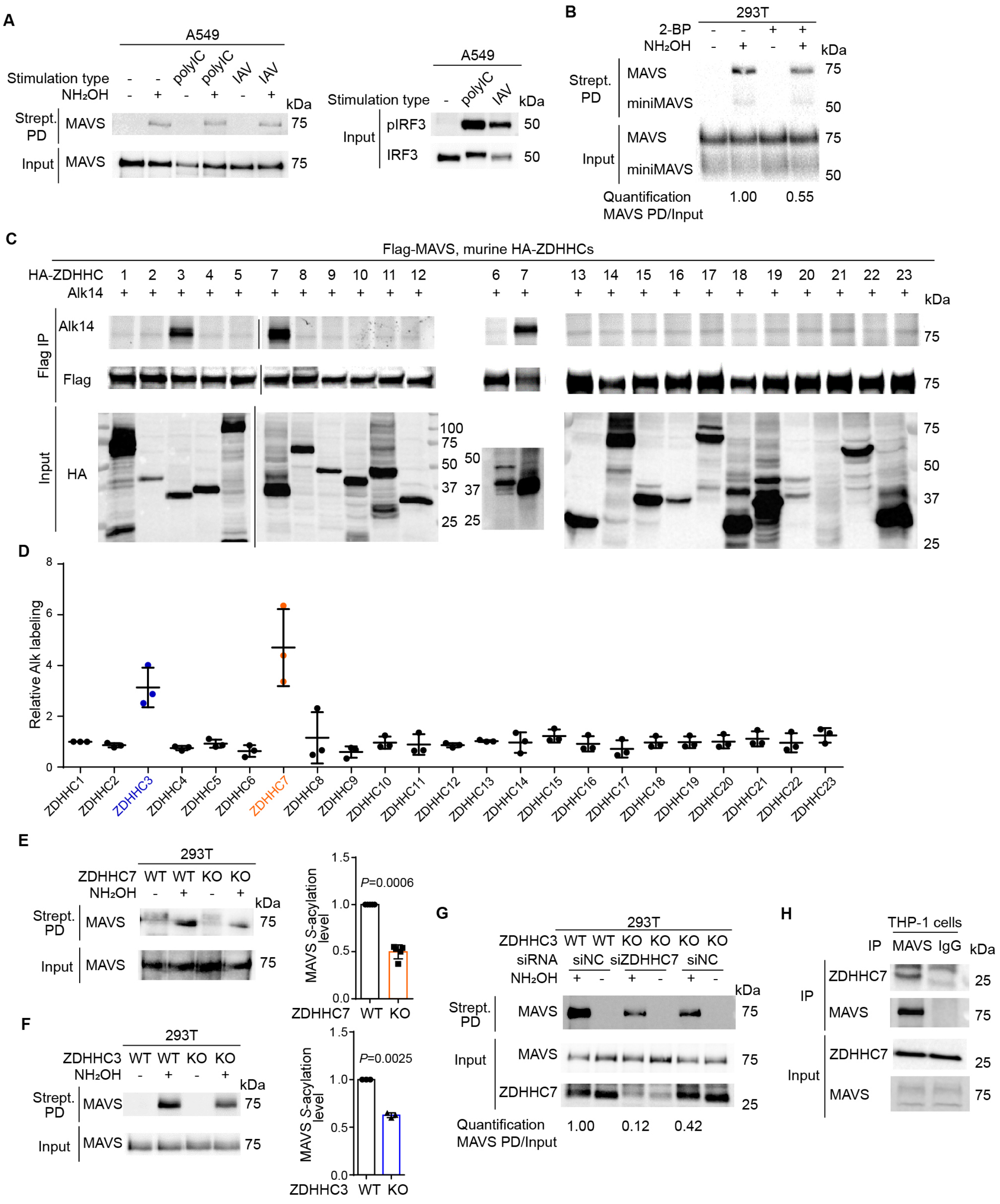

Fig. S1. Identification and characterization of MAVS *S*-palmitoylation. (*A*) Endogenous *S*-acylation of MAVS was not affected by RLR signaling activation. Endogenous MAVS *S*-acylation in A549 cells without stimulation, with 2 μg/mL poly(I:C) treatment for 6 h, or with IAV infection was analyzed by ABE assay. Strept PD signal on the immunoblots represented 50% of MAVS *S*-acylation level and the input represented 5% of total MAVS protein. (*B*) 2-BP inhibited MAVS *S*-acylation. Endogenous *S*-acylation of MAVS and miniMAVS was detected in HEK293T cells treated with 50 μM 2-BP or DMSO for 6 h using ABE assay. (*C*) Identification of palmitoyltransferases of MAVS by

ZDHHC screening. HEK293T cells were transfected with Flag-MAVS and HA-ZDHHC1-23. The palmitoylation level was detected by Alk14 metabolic labeling, click chemistry for the installation of TAMRA, and subsequent detection via in-gel fluorescence. (*D*) Quantification of the relative palmitoylation levels in *C*. Number of independent experiments, n = 3. (*E*) *ZDHHC7* knockout in HEK293T cells decreased endogenous MAVS *S*-palmitoylation. Quantification of MAVS *S*-acylation levels of five independent experiments of ABE analysis in WT and *ZDHHC7*$^{-/-}$ 293T cells, n = 5. (*F*) *ZDHHC3* knockout in HEK293T cells decreased endogenous MAVS *S*-palmitoylation. Endogenous *S*-acylation of MAVS was detected in WT or *ZDHHC3*$^{-/-}$ 293T cells using ABE assay. Quantification of MAVS *S*-acylation levels of three independent experiments is shown on the right, n = 3. (*G*) ZDHHC3 and ZDHHC7 are the two major palmitoyltransferases of MAVS. ABE analysis of endogenous MAVS *S*-acylation in WT, *ZDHHC7* knockdown *ZDHHC3*$^{-/-}$ 293T, and control knockdown *ZDHHC3*$^{-/-}$ 293T cells. (*H*) MAVS co-IP with ZDHHC7 in THP-1 cells. THP-1 cells were stimulated with PMA overnight and subjected to immunoprecipitation of MAVS. *P* values were calculated using two-tailed ratio paired *t*-tests in *E* and *F*.

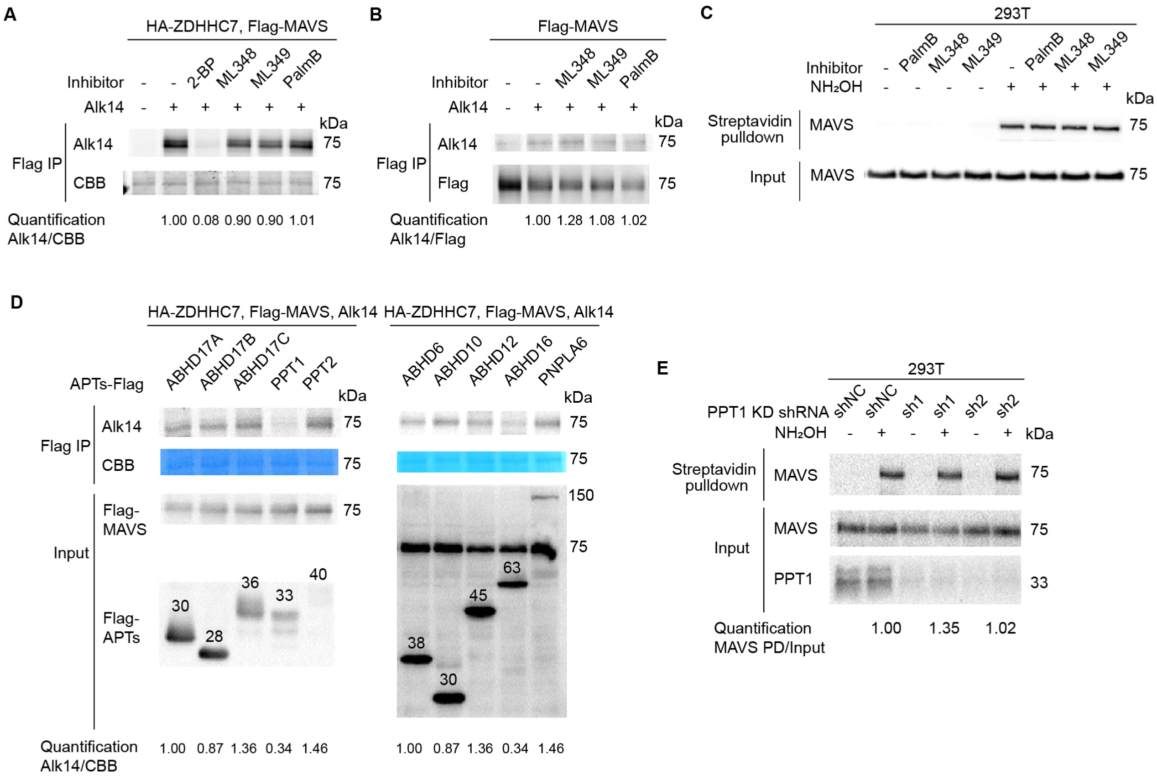

Fig. S2. Identification of the depalmitoylase(s) of MAVS. (*A*) ZDHHC7-catalyzed MAVS palmitoylation was not increased by depalmitoylase inhibitors. HEK293T cells transfected with Flag-MAVS and HA-ZDHHC7 were treated with 2-BP, APT1 inhibitor ML348, APT2 inhibitor ML349, and pan-depalmitoylase inhibitor Palmostatin B (PalmB). Alk14 labeling and click chemistry were performed. Palmitoylation was detected by in-gel fluorescence. (*B*) MAVS palmitoylation did not increase by treatment of depalmitoylase inhibitors. HEK293T cells transfected with Flag-MAVS were treated with ML348, ML349 or PalmB. Method of palmitoylation detection was the same as in *A*. (*C*) Endogenous *S*-acylation of MAVS was not affected by depalmitoylase inhibitors. ABE was performed in HEK293T cells treated with ML348, ML349 or PalmB. (*D*) PPT1 overexpression decreased MAVS palmitoylation. HEK293T cells transfected with Flag-MAVS and HA-ZDHHC7 were co-transfected with the indicated acyl-protein thioesterases. Method of palmitoylation detection was the same as in *A*. (*E*) *S*-acylation of MAVS was not affected by *PPT1* knockdown. ABE was performed in *PPT1* stably knocked-down HEK293T cells using shRNA 1 or 2, or control shRNA (shNC). The levels of MAVS palmitoylation were quantified by in-gel fluorescence signal divided by CBB intensity in *A*, *B* and *D*.

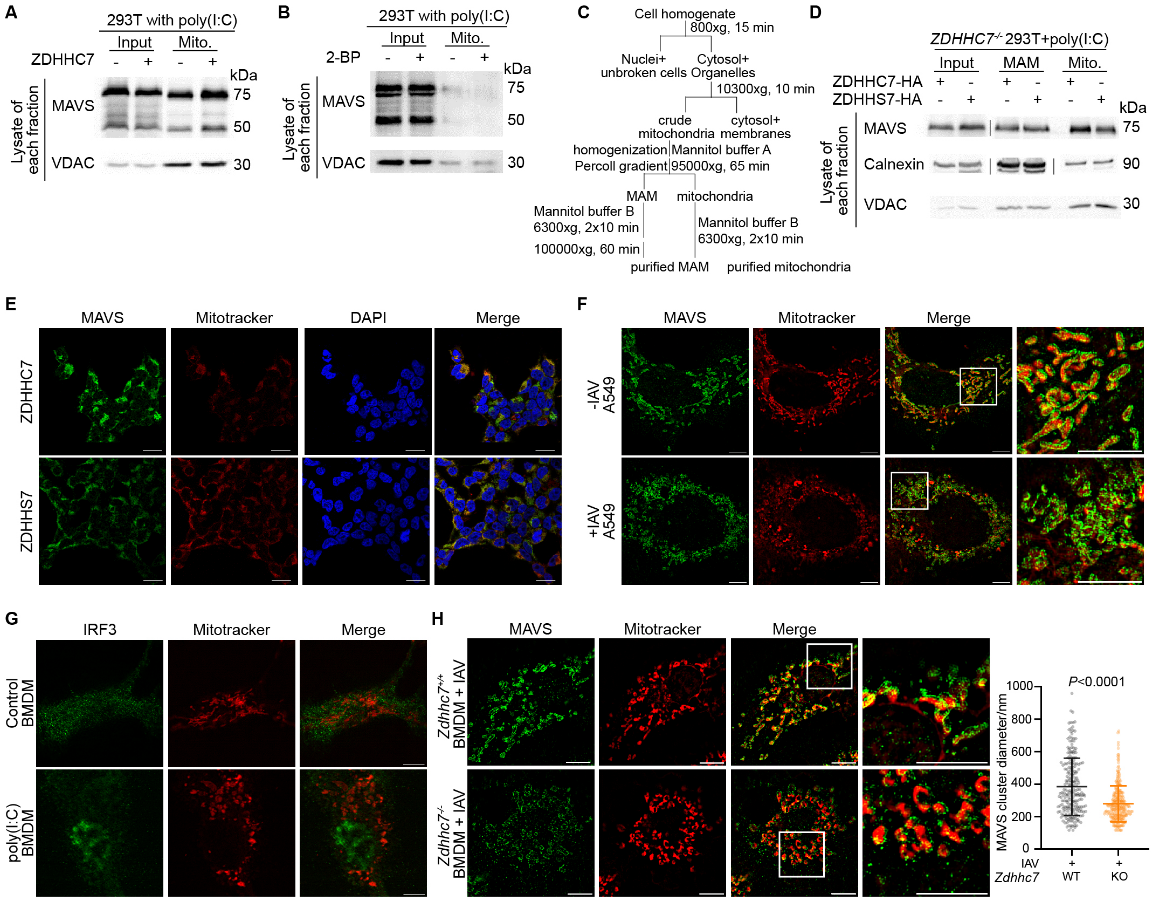

Fig. S3. ZDHHC7 palmitoylates MAVS to stabilize its aggregation. (*A*, *B*) MAVS palmitoylation enhanced its localization on the mitochondria upon RLR activation. HEK293T cells transfected with or without HA-ZDHHC7(*A*) or treated with or without 2-BP (*B*) were stimulated with 2 μg/mL poly(I:C) by transfection for 8 h and underwent subcellular fractionation. The levels of MAVS and VDAC in the mitochondria fraction and the cell lysate were analyzed by immunoblotting. (*C*) A schematic illustration of the procedure for isolation and purification of the mitochondria-associated ER membrane and the mitochondria using ultracentrifugation. (*D*) The effect of *S*-palmitoylation on the mitochondria and MAM distribution of MAVS upon activation. *ZDHHC7*$^{-/-}$ 293T cells reintroduced with HA-ZDHHC7 or HA-ZDHHS7 were treated with 2 μg/mL poly(I:C) by transfection for 8 h and were subjected to Percoll gradient as described in *C*. The distribution of MAVS, Calnexin (ER marker) and VDAC was analyzed by immunoblotting. (*E*) MAVS showed more intense signal on the mitochondria with ZDHHC7 overexpression. *ZDHHC7*$^{-/-}$ 293T cells reintroduced HA-ZDHHC7 or HA-ZDHHS7 were treated with 2 μg/mL poly(I:C) by transfection for 8 h. Endogenous MAVS (green), mitochondria (red), and nucleus (blue) were visualized using confocal microscopy. Scale bars, 30 μm. (*F*) MAVS redistributed and aggregated after IAV infection. Endogenous MAVS (green) and mitochondria (red) of A549 infected with or without IAV for 12 h were visualized using SR-SIM. Scale bars, 5.0 μm. (*G*) Poly(I:C) stimulation induced mitochondrial fission. Distribution of IRF3 (green) and morphology of the mitochondria of non-

stimulated or poly(I:C)-transfected BMDMs were imaged using SR-SIM. Scale bars, 5.0 μm. (*H*) MAVS exhibited less aggregation in *Zdhhc7*$^{-/-}$ BMDMs. WT and *Zdhhc7*$^{-/-}$ BMDMs were infected with IAV for 8 h. Endogenous MAVS (green) and mitochondria (red) were visualized by SR-SIM. Scale bars, 5.0 μm. The diameter of MAVS clusters was measured from the SR-SIM images of IAV infected WT and *Zdhhc7*$^{-/-}$ BMDMs. WT, n = 230; *Zdhhc7*$^{-/-}$, n = 279.

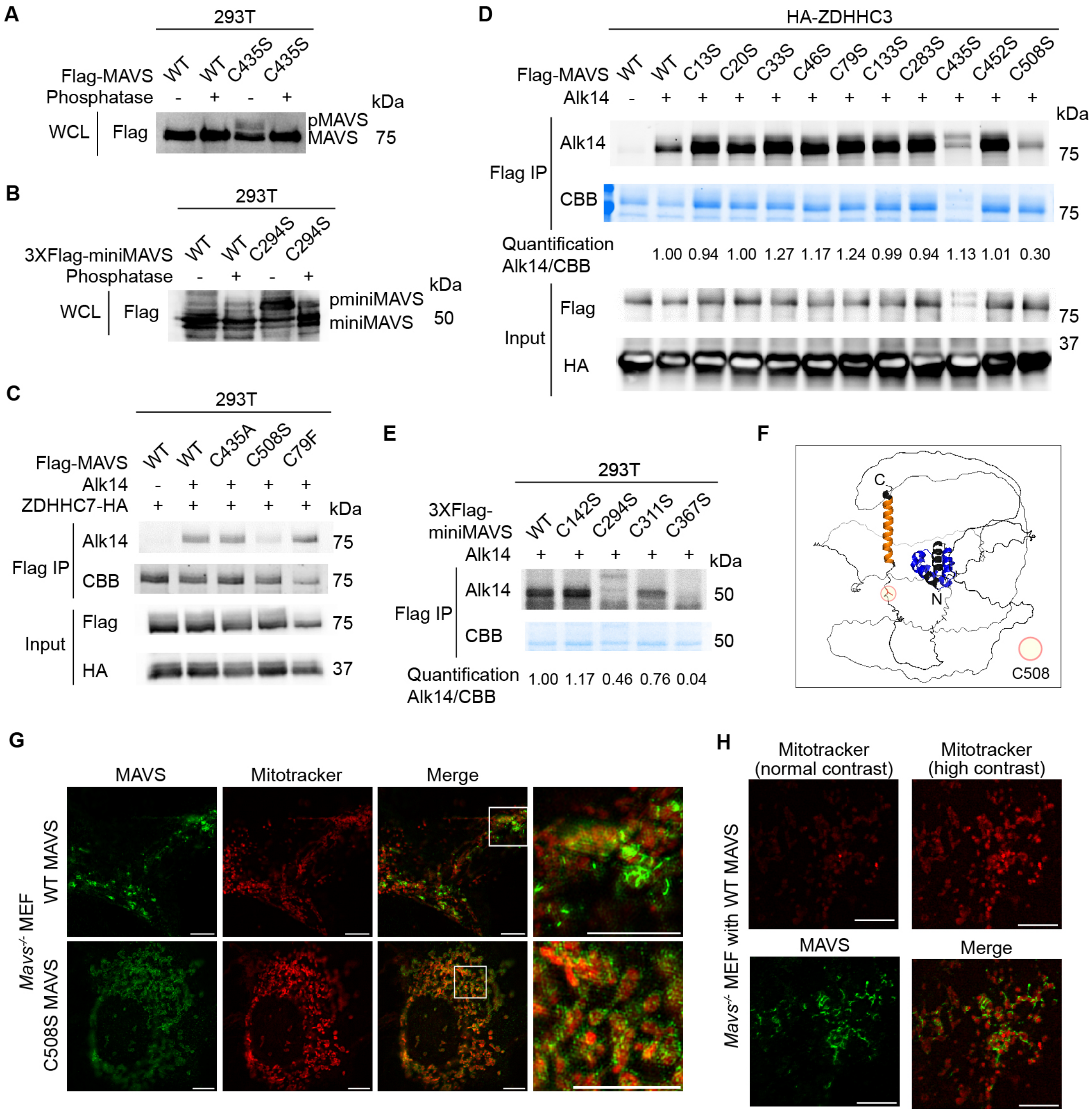

Fig. S4. *S*-palmitoylation on C508 promotes MAVS aggregation. (*A*, *B*) MAVS C435S mutant was phosphorylated. HEK293T cells were transfected with WT or C435S mutant Flag-MAVS (*A*) or transfected with WT or C294S mutant 3xFlag-miniMAVS (*B*) for 24 h. Cell lysate was treated with or without phosphatase and subjected to immunoblotting. MiniMAVS C294 corresponds to full-length MAVS C435. (*C*) MAVS C79F did not decrease MAVS palmitoylation catalyzed by ZDHHC7. HEK293T cells transfected with WT, C435A, C508S or C79F MAVS with HA-ZDHHC7. The palmitoylation signals were analyzed by in-gel fluorescence. (*D*) Identification of the *S*-palmitoylation site of MAVS with ZDHHC3 overexpression. HEK293T cells were transfected with WT Flag-MAVS or indicated Cys-to-Ser mutant and HA-ZDHHC3. Palmitoylation signal was detected by in-gel fluorescence after Flag-MAVS immunoprecipitation and click chemistry. C435S mutant MAVS appeared in two bands due to its phosphorylation. (*E*) HEK293T cells transfected with WT, C142S, C294S, C311S or C367S mutant 3xFlag-miniMAVS were metabolically labelled with 50 μM Alk14 for 6 h. The palmitoylation signal was detected by in-gel fluorescence. MiniMAVS

C142, C294, C311, and C367 correspond to full-length MAVS C283, C435, C452, and C508, respectively. (*F*) AlphaFold predicted MAVS structure with C508 highlighted. The CARD domain of MAVS is labeled blue, and the TM domain is labeled orange. (*G*) WT but not C508S mutant MAVS showed redistribution and aggregation when activated. MAVS (green) and mitochondria (red) in poly(I:C) transfected *Mavs*$^{-/-}$ MEF cells stably expressing WT or C508S MAVS were visualized using SR-SIM. Scale bars, 5.0 μm. (*H*) WT MAVS showed hyper aggregation with poly(I:C) stimulation. MAVS (green) and mitochondria (red) in poly(I:C) transfected *Mavs*$^{-/-}$ MEF cells stably expressing WT MAVS were visualized using SR-SIM. Scale bars, 5.0 μm. The contrast of the MitoTracker (red) channel was adjusted for a better presentation of mitochondria. The levels of MAVS palmitoylation were quantified by in-gel fluorescence signal divided by CBB intensity in *D* and *E*.

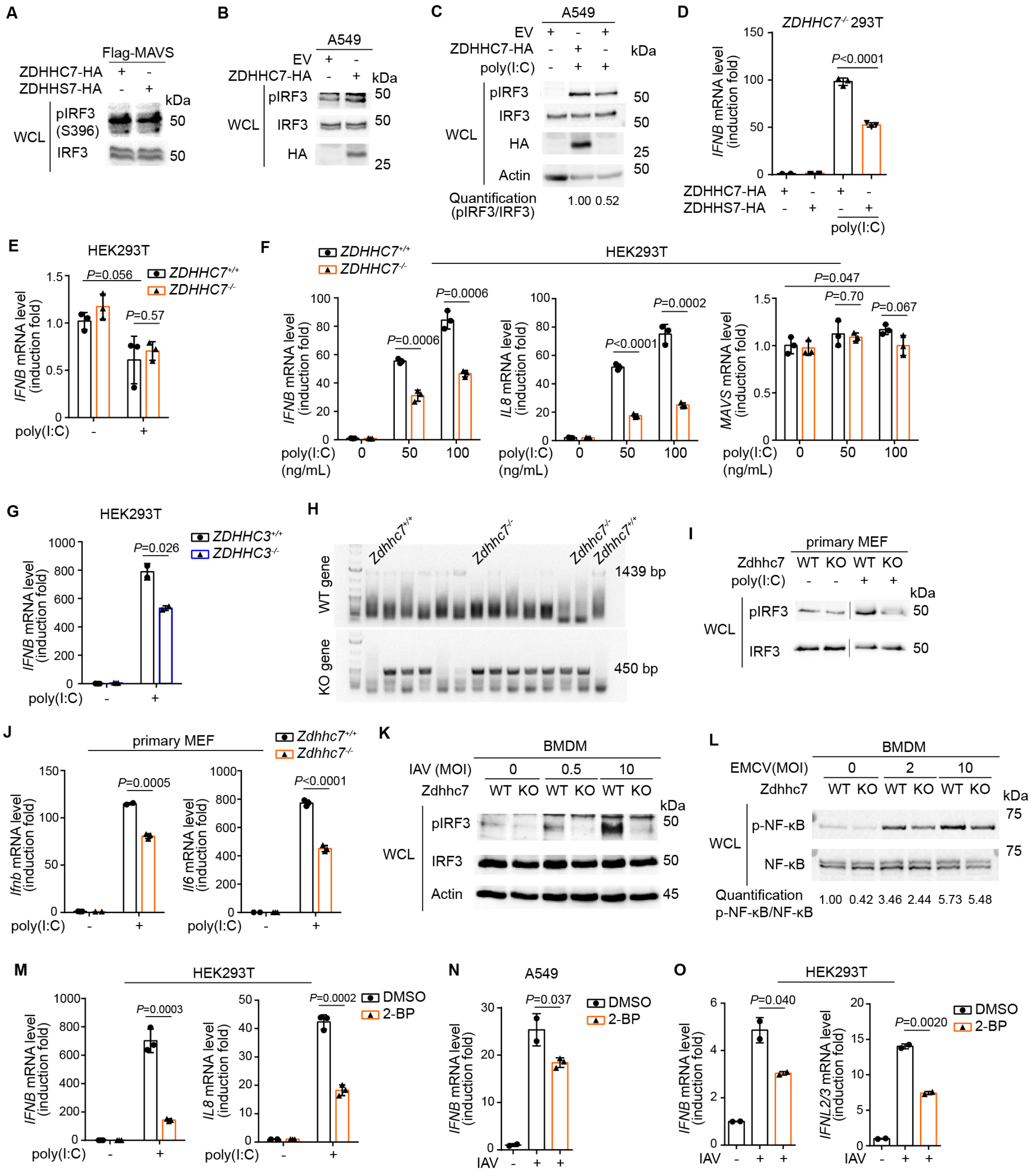

Fig. S5. ZDHHC7 enhances RLR signaling and antiviral immune response. (*A*) ZDHHC7 but not ZDHHS7 increased pIRF3 induced by MAVS overexpression. Immunoblots of pIRF3 and IRF3 in the whole cell lysate of HEK293T cells transfected with Flag-MAVS and HA-ZDHHC7 or HA-ZDHHS7. (*B*, *C*) Overexpression of ZDHHC7 increases IRF3 activation at steady state (*B*) or with RLR activation by poly(I:C) transfection (*C*). A549 cells were transfected with ZDHHC7 for 24 h. Activation of IRF3 was evaluated by immunoblots of pIRF3. (*D*) ZDHHC7 reintroduced in *ZDHHC7*$^{-/-}$ HEK293T cells enhanced RLR activation. qPCR analysis of the induction of *IFNB* in the HA-ZDHHC7 or HA-ZDHHS7 transfected HEK293T cells stimulated by poly(I:C). (*E*)

HEK293T cells did not respond to TLR3 stimulation. WT and *ZDHHC7*$^{-/-}$ HEK293T cells were treated with or without 200 ng/mL poly(I:C) by direct addition for 16 h. Induction of *IFNB* was measured using qPCR. (*F*) *ZDHHC7* knockout decreased RLR signaling activation. WT and *ZDHHC7*$^{-/-}$ HEK293T were stimulated with 0, 50, 100 ng/mL poly(I:C) by transfection for 16 h. Induction of *IFNB*, *IL8* and *MAVS* was measured using qPCR. (*G*) *ZDHHC3* knockout decreased RLR signaling activation. WT and *ZDHHC3*$^{-/-}$ HEK293T cells were stimulated with or without 200 ng/mL poly(I:C) by transfection for 16 h. Induction of *IFNB* was measured using qPCR. (*H*) Genotyping of 14 embryos from *Zdhhc7*$^{+/-}$ crossed with *Zdhhc7*$^{+/-}$ mice. WT gene PCR resulted in a 1439 bp product. KO gene PCR resulted in a 450 bp product. Embryos determined as *Zdhhc7*$^{+/+}$ or *Zdhhc7*$^{-/-}$ was labeled on the top. The unlabeled ones were heterozygous or uncertain. (*I*) *Zdhhc7* KO decreased IRF3 activation by poly(I:C) transfection. WT or Z*dhhc7*$^{-/-}$ primary MEFs were stimulated with or without 200 ng/mL poly(I:C) for 16 h and then blotted for pIRF3 and IRF3. (*J*) Zdhhc7 enhanced the expression of type I interferon and pro-inflammatory cytokine. Induction of *Ifnb* and *Il6* in poly(I:C) stimulated WT or Z*dhhc7*$^{-/-}$ primary MEFs was analyzed by qPCR. (*K*) Zdhhc7 strengthened anti-IAV response at different MOIs. WT and *Zdhhc7*$^{-/-}$ primary BMDMs were infected by IAV at 0, 0.5 and 10 MOI for 8 h. Immunoblots of pIRF3 (Ser396), IRF3, and Actin were examined to assess the antiviral response. (*L*) *ZDHHC7* knockout suppressed the activation of NF-κB. WT or *Zdhhc7*$^{-/-}$ BMDMs were infected with EMCV at the indicated doses for 6 h. Activation of NF-κB signaling was assessed using immunoblots for p-NF-κB. (*M*) 2-BP inhibited RLR signaling. HEK293T cells treated with or without 50 μM 2-BP were stimulated by 200 ng/mL poly(I:C) transfection for 16 h. Induction of *IFNB* and *IL8* was measured by qPCR. (*N*) 2-BP inhibited type I interferon production in A549 cells with IAV infection. Induction of *IFNB* of IAV infected A549 treated with 2-BP or DMSO was analyzed by qPCR. (*O*) 2-BP inhibited type I and type III interferon production in HEK293T cells with IAV infection. Induction of *IFNB* and *IFNL2/3* in IAV infected HEK293T cells treated with 2-BP or DMSO was analyzed by qPCR. mRNA level in each sample was normalized to internal control (*ACTB* or *Actb*). *P* values were calculated using two-tailed unpaired *t*-tests. Data are mean ± SD.

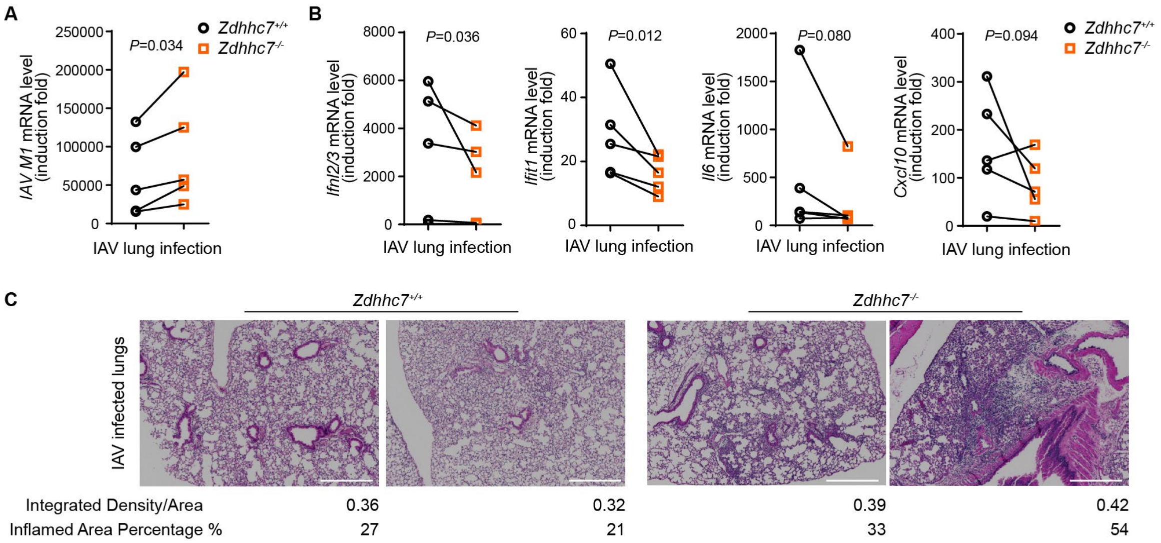

Fig. S6. ZDHHC7 facilities antiviral immune response in vivo. *Zdhhc7*$^{-/-}$ mice had weaker antiviral immune response compared to WT. qPCR measurement of IAV *M1* mRNA level (*A*) and induction of *Ifnl2/3*, *Ifit1*, *Il6*, and *Cxcl10* (*B*) in the lungs of WT and *Zdhhc7*$^{-/-}$ mice that intranasally infected with 1000 pfu of IAV H1N1 strain A/PR/8/34 for 48 h. Each pair of WT and *Zdhhc7*$^{-/-}$ lung samples represents an independent biological repeat of the IAV infection mouse experiment. Number of independent experiments, n = 5. mRNA level was normalized to internal control (*Actb*). *P* values were calculated using two-tailed ratio paired *t*-tests. (*C*) Representative H&E staining of the lung sections of 48 h-IAV infected WT and *Zdhhc7*$^{-/-}$ mice. Scale bars, 500 μm. Integrated density/area and inflamed area percentage were quantified in each image.

Table S1. Sequence of oligoes for molecular cloning, genotyping and qPCR

Primer Name	Sequence (5' to 3')	Purpose
MAVS_C13S_For	CAAGACCTATAAGTATATCAGCCGCAATTTCAGC	Mutagenesis
MAVS_C13S_Rev	GCTGAAATTGCGGCTGATATACTTATAGGTCTTG	
MAVS_C20S_For	GCCGCAATTTCAGCAATTTTAGCAATGTGGATGTTG	
MAVS_C20S_Rev	CAACATCCACATTGCTAAAATTGCTGAAATTGCGGC	
MAVS_C33S_For	CTTACCTGCCCAGCCTCACAGCAAG	
MAVS_C33S_Rev	CTTGCTGTGAGGCTGGGCAGGTAAG	
MAVS_C46S_For	GACTGCGGGCCACCAGCACACTCTCAGGGAAC	
MAVS_C46S_Rev	GTTCCCTGAGAGTGTGCTGGTGGCCCGCAGTC	
MAVS_C79S_For	GCGGCACTGAGGGGCTCTGAGCTAGTTGATCTC	
MAVS_C79S_Rev	GAGATCAACTAGCTCAGAGCCCCTCAGTGCCGC	
MAVS_C133S_For	CCCCTACAACAGCAGCAGAGAGAAGG	
MAVS_C133S_Rev	CCTTCTCTCTGCTGCTGTTGTAGGGG	
MAVS_C283S_For	GCCGAGCCTATCATCAGCTCCAGTGGGGCAGAG	
MAVS_C283S_Rev	CTCTGCCCCACTGGAGCTGATGATAGGCTCGGC	
MAVS_C435S_For	CGTTCTCGGGCAGCTTCGAGGATCTTG	
MAVS_C435S_Rev	CAAGATCCTCGAAGCTGCCCGAGAACG	
MAVS_C452S_For	GGCATGGGGCCCAGCCATGGCCCAG	
MAVS_C452S_Rev	CTGGGCCATGGCTGGGCCCCATGCC	
MAVS_C508S_For	GAGAGGGAGGTGCCAAGCCACAGGCCCTCAC	
MAVS_C508S_Rev	GTGAGGGCCTGTGGCTTGGCACCTCCCTCTC	
MAVS_C435A_For	CGTTCTCGGGCGCCTTCGAGGATCTTG	
MAVS_C435A_Rev	CAAGATCCTCGAAGGCGCCCGAGAACG	
MAVS_C79F_For	GCGGCACTGAGGGGCTTTGAGCTAGTTGATCTC	
MAVS_C79F_Rev	GAGATCAACTAGCTCAAAGCCCCTCAGTGCCGC	
ACTIN-For	CACCATTGGCAATGAGCGGTTC	qPCR
ACTIN-Rev	AGGTCTTTGCGGATGTCCACGT	

IFN-β-For	CTTGGATTCCTACAAAGAAGCAGC	
IFN-β-Rev	TCCTCCTTCTGGAACTGCTGCA	
IL-8-Fev	GAGAGTGATTGAGAGTGGACCAC	
IL-8-Rev	CACAACCCTCTGCACCCAGTTT	
RIGI-For	AGCGGCGGAACCTGCTGGCC	
RIG1-Rev	TCTTTAAAGCATCCACAAGT	
MAVS-Fev	GTCACTTCCTGCTGAGA	
MAVS-Rev	TGCTCTGAATTCTCTCCT	
IFNL2/3-Fev	CAGCTGCAGGTGAGGGA	
IFNL2/3-Rev	GCGGTGGCCTCCAGAACCTT	
IAV M1-Fev	TCAGGCCCCCTCAAAGCCGA	
IAV M1-Rev	GGGCACGGTGAGCGTGAACA	
Actin-For (mouse)	CGTGAAAAGATGACCCAGATCA	
Actin-Rev (mouse)	CACAGCCTGGATGGCTACGT	
Mavs-For (mouse)	CTGCCAACACAATACCACCTGAG	
Mavs-Rev (mouse)	TCTCTGGTCCAGAGTGCAAGCT	
Ifnl2/3-For (mouse)	AGCTGCAGGCCTTCAAAAAG	
Ifnl2/3-Rev (mouse)	TGGGAGTGAATGTGGCTCAG	
Ifnb-For (mouse)	CCAAGAAAGGACGAACATTCG	
Ifnb-Rev (mouse)	TCCGTCATCTCCATAGGGATCT	
Ifit1-For (mouse)	GAACCCATTGGGGATGCACAACCT	
Ifit1-Rev (mouse)	CTTGTCCAGGTAGATCTGGGCTTCT	
Cxcl10-For (mouse)	CATCCCTGCGAGCCTATCC	
Cxcl10-Rev (mouse)	CATCTCTGCTCATCATTCTTTTTCA	
Il6-For (mouse)	TACCACTTCACAAGTCGGAGGC	
Il6-Rev (mouse)	CTGCAAGTGCATCATCGTTGTTC	
EMCV-For	GACGCTTGAAGACGTTGTCTTCTTA	
EMCV-Rev	CCCTACCTCACGGAATGGGGCAAAG	
Rig-1-For (mouse)	AGCCAAGGATGTCTCCGAGGAA	
Rig-1-Rev (mouse)	ACACTGAGCACGCTTTGTGGAC	
Zdhhc7_WT-For	TGAGCCAGGATGGATTTCAGACA	Genotyping

Zdhhc7_WT-Rev	TGCCCTCGGACGCAGGAGATGAA	
Zdhhc7_KO-For	TCCCCTGATGTATGCGAATGTCC	
Zdhhc7_KO-Rev	AACAGGTGCCTTTTGAATGTCAG	
